# Inferring speciation modes in a clade of Iberian chafers from rates of morphological evolution in different character systems

**DOI:** 10.1186/1471-2148-9-234

**Published:** 2009-09-15

**Authors:** Dirk Ahrens, Ignacio Ribera

**Affiliations:** 1 Zoologische Staatssammlung München, Münchhausenstr. 21, 81247 Munich, Germany; 2 Department of Entomology, Natural History Museum, Cromwell Road, London SW7 5BD, UK; 3 Museo Nacional de Ciencias Naturales, José Gutiérrez Abascal 2, 28006 Madrid, Spain; 4 Institute of Evolutionary Biology (CSIC-UPF), Passeig Maritim de la Barceloneta 37-49, 08003 Barcelona, Spain

## Abstract

**Background:**

Studies of speciation mode based on phylogenies usually test the predicted effect on diversification patterns or on geographical distribution of closely related species. Here we outline an approach to infer the prevalent speciation mode in Iberian Hymenoplia chafers through the comparison of the evolutionary rates of morphological character systems likely to be related to sexual or ecological selection. Assuming that mitochondrial evolution is neutral and not related to measured phenotypic differences among the species, we contrast hypothetic outcomes of three speciation modes: 1) geographic isolation with subsequent random morphological divergence, resulting in overall change proportional to the mtDNA rate; 2) sexual selection on size and shape of the male intromittent organs, resulting in an evolutionary rate decoupled to that of the mtDNA; and 3) ecological segregation, reflected in character systems presumably related to ecological or biological adaptations, with rates decoupled from that of the mtDNA.

**Results:**

The evolutionary rate of qualitative external body characters was significantly correlated to that of the mtDNA both for the overall root-to-tip patristic distances and the individual inter-node branches, as measured with standard statistics and the randomization of a global comparison metric (the z-score). The rate of the body morphospace was significantly correlated to that of the mtDNA only for the individual branches, but not for the patristic distances, while that of the paramere outline was significantly correlated with mtDNA rates only for the patristic distances but not for the individual branches.

**Conclusion:**

Structural morphological characters, often used for species recognition, have evolved at a rate proportional to that of the mtDNA, with no evidence of directional or stabilising selection according to our measures. The change in body morphospace seems to have evolved randomly at short term, but the overall change is different from that expected under a pure random drift or randomly fluctuating selection, reflecting either directional or stabilising selection or developmental constraints. Short term changes in paramere shape possibly reflect sexual selection, but their overall amount of change was unconstrained, possibly reflecting their lack of functionality. Our approach may be useful to provide indirect insights into the prevalence of different speciation modes in entire lineages when direct evidence is lacking.

## Background

The study of speciation patterns has been traditionally dominated by the role of geography [1,2], most likely due to the ready availability of species-level phylogenies and data on geographical ranges of species [3,4]. Other speciation modes, such as sexual selection or ecological segregation, have usually been studied with phylogenies through the influence on species numbers or diversification rates [5-7], the evolution of ecomorphological characters in relation to habitat type and co-occurrence [8], the evolution of sexual characters [9-11], or the reconstruction of the ancestral niche [12]. However, there are only few cases in which the prevalence of these three main speciation modes (geographical isolation, sexual selection, ecological segregation) has been assessed in a comparative framework, rather than testing their individual effect on a target group (but see e.g., [13,14]).

In this work we use different morphological traits of a group of chafers of the genus *Hymenoplia *Eschscholtz to elucidate the prevalent speciation mode. The genus *Hymenoplia *is part of the tribe Sericini, a group of chafer beetles (Scarabaeidae), which are particularly diverse among the phytophagous scarabs (Pleurosticti) [15]. Species of *Hymenoplia *are ecologically and morphologically very similar (see Methods), and their ranges have a large degree of overlap at larger geographical scales, although they generally do not co-occur at the local level. This pattern suggests either that there could be factors promoting speciation other than geographical isolation, or that the geographical signature of speciation has been lost by post-speciation range movements [16]. Through the use of species-level phylogenies with multiple samples per species and different sets of morphological traits, we aim to detect the evidence for the influence of selection during trait evolution in order to discriminate between the dominant role of three general modes of speciation: geographical, ecological and sexual, taking into account infraspecific variation. Our null assumption is that if the rate of evolution of a character system across the phylogeny is proportional to time it could be considered "neutral", in the sense that it is not subjected to any strong directional or stabilizing selection ([17], see other examples in e.g. [1] for genital shape; [18] for ecological differences; [19] for general morphological traits).

To have a baseline for a neutral rate we use that of the mitochondrial DNA (mtDNA) [17,20,21]), and consider it proportional to time (despite possible exceptions, see Discussion), and not related to the phenotypic expression of any of the measured morphological characters. We compare this reference neutral evolutionary rate with that of three different morphological character systems: 1) shape of male genitalia as indicative of sexual selection; 2) general body size and shape and 3) morphological structural characters (including those used for species recognition), the last two indicative of a possible ecological partitioning. The character systems that show an evolutionary rate significantly correlated to that of the mtDNA could be said to be neutral and proportional to time, i.e. evolving under a Brownian motion model [22] or subjected to random fluctuating selection [23]. Those differing significantly could be said to have evolutionary rates not proportional to time, likely to be due to stabilizing or directional selection [23]. Specifically, we examine three hypotheses:

1) Geographical speciation: if the dominant mode of speciation in the group has been geographical isolation with subsequent drift for both molecular and morphological characters (e.g., [1]), all character sets could be expected to evolve in a predominantly neutral mode, and in consequence the rates of change be correlated among them as a result of their own correlation with time or to general pleiotropic effects.

2) Speciation driven by sexual selection (through divergence in shape and size of the genital structures): this should be reflected in the rates of evolution of the morphology of the intromittent portions of copulatory organs [9,24]. If their shape and size are subjected to selection, their rates of evolution will not be proportional to time or its surrogate, the rates of mtDNA change. Given the very weak sexual dimorphism in species of *Hymenoplia*, only apparent by enlarged and lobiform anterior inner protarsal claws in the male, it may be expected that sexual selection did not have an impact on structural morphology or body shape.

3) Ecological segregation: although there is no information on the detailed ecology and biology of most species of *Hymenoplia*, it seems reasonable to assume that these potential differences could be reflected either in body shape and size or in structural external characters. Again, if these are subjected to selection, it is expected that their evolutionary rates will be de-coupled to that of the mitochondrial genome.

The decoupling of the molecular and morphological rates of evolution could be interpreted as indicative of the presence of directional or stabilizing selection. However, different types of selection are expected to result in contrasting patterns of morphological variation [23]. Diversifying or directional selection should result in increasing divergence between species over evolutionary time, with a low ratio of intra- to interspecific variation [25]. On the contrary, stabilizing selection may result in the random drift of the morphological change within some fixed boundaries [26-28]. In the later, rates of morphological evolution should be neutral and proportional to time within the allowed boundaries, but over longer evolutionary periods this relationship is not maintained, and rates should be decoupled from time (i.e. mtDNA rates) [17]. Any sign of selection on the shape of the male copulatory organs is likely to be related to sexual selection and speciation, but the presence of selection (i.e. decoupling from neutral rates) in body morphospace or other morphological characters could be related to the same speciation process or to subsequet anagenetic evolution.

The comparisons of rates of change are performed at two levels [29,30]: 1) Individual branches, i.e. internodal distances. We compare the change of each character system over all corresponding individual branches of the estimated topology. This gives an overall relationship of relative rates of change for multiple individual time periods. 2) Patristic distances between terminals. We computed a patristic distance matrix between all terminals for each character system, and then compared their association with the mtDNA distances through multiple Mantel tests. We also use pairwise plots of patristic distances to test for general differences between infraspecific and interspecific variation. Although our methods for hypothesis testing do not require the a-priori delimitation of species, which in groups with a complex taxonomy (like the genus *Hymenoplia*) is not a trivial issue, evolutionary processes may be expected to be different at infra and interspecific level under certain types of selection. The type of molecular data used (mitochondrial DNA) and the difficulties in the taxonomy of the group (see below) prevents the use of methods requiring the precise delimitation of species (e.g. Fontaneto et al. 2007), but the comparisons of the slopes of the regressions of intra- and among-species variation could provide insights into how variation is partitioned.

There is an extensive literature on the relationship between morphological and molecular evolutionary rates (e.g., [17,19,23,25,30-33]), although in most cases the rates were compared across different phylogenies, not between different morphological character systems in the same phylogenetic tree. By the use of parallel comparisons of morphological characters measured in the same specimens across the same phylogeny we avoid many of the problems associated with previous approaches (such as e.g. differences in taxon sampling, or the evolutionary background or the biology of the species; see [33] for a recent review). The existence of clear differences in the evolutionary rates among the studied character systems will provide insightful suggestions as to which has been the dominant speciation mode in this group of polyphagous scarab chafers, and thus contribute to understand which could have been the key factors for their diversification.

**Figure 1:**
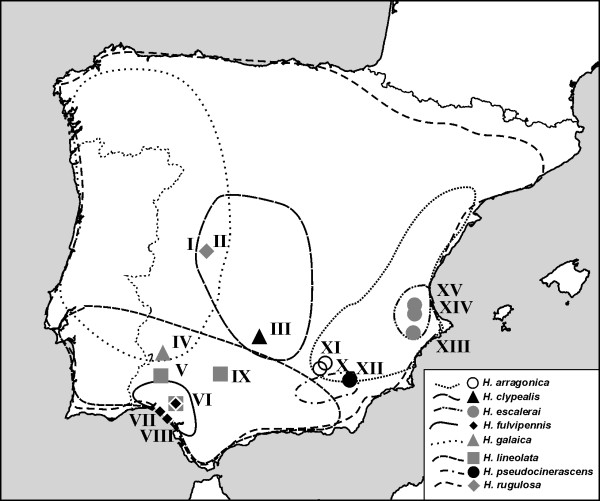
**Sample localities of the species studied (I-XV) including approximated range extensions of the species studied according to Baguena Corella (1967) and Baraud (1992)**.

Species of *Hymenoplia *feed preferably on leaves and inflorescences of grasses as adults [34,35] and presumably on humus and roots in the larval stages, like other members of the Pleurosticts [36]. A recent phylogenetic analysis [37] showed that *Hymenoplia*, with ca. 46 taxa, is part of the least diverse (ca. 150 taxa) of the two sericine sister lineages of the Old World (with a combined diversity of ca. 3,200 species). *Hymenoplia *species occur exclusively in the Western-Mediterranean region, and a third of them in the Iberian peninsula [38]. The traditional taxonomy of the genus *Hymenoplia *has been confusing, due to the lack of clear diagnostic characters, the variability among and within populations, and their largely overlapping geographical ranges [39,40]. They do have similar ecologies, but, intriguingly, despite the general sympatry of their ranges (Figure 1), they very rarely co-occur syntopically in the same locality [35,39]

## Methods

### Taxon sampling, DNA extraction and identification

*Hymenoplia *specimens were collected from grasses at 16 sites in the southern Iberian peninsula (Figure 1) in 2006. Among the hundreds of *Hymenoplia *specimens sampled by the first author from a vast range of study sites throughout all southern Spain (Figure 1), in only one site was more than one species found (D. Ahrens, unpublished data). Genomic DNA was extracted from thoracic muscle tissue with Charge Switch gDNA micro tissue kit (Invitrogen, Paisley, UK). Extracted specimens were dry mounted with minimal damage, allowing further morphological investigation. Vouchers are deposited in the D. Ahrens collection (NHM). Diagnostic characters to distinguish adult morphospecies were those traditionally used in taxonomic studies of the group, including body size and shape, coloration, surface sculpture and pilosity as well as male genital morphology [39-41]. All identifications were validated by comparison with the type specimens preserved in the collection of the MNCN. A representative subset of 38 male individuals of the eight named species for which we could obtain DNA samples (half of known Iberian *Hymenoplia *species [38]) plus one outgroup species (Additional file 1) were used for DNA sequencing and phylogenetic reconstruction.

### Morphological characters and morphometric measurements

We studied three different morphological character systems:

1) General structural characters, including mainly features of cuticular integument such as body surface texture, pilosity and color, but also fine structures of legs and tarsomeres (Additional file 2), which are among those used for the species recognition. Nineteen discrete characters were scored for each adult male individual (Additional files 3, 4). The variation of structural morphological characters was visually checked based on a multiple scaling analysis performed in XLSTAT 2007 (Addinsoft, Paris) using the absolute morphological distances calculated from the original data in PAUP*4.0b10 [42] (Additional file 5).

2) Morphometric characterization of the body shape and size. To characterize the morphospace defined by the studied species we measured total length, width of pronotum, length of elytra and width of elytra (Additional file 6). Due to the limited size range of the studied samples it was not necessary to transform the data, and we used the raw measurements for the analyses to obtain the matrix of Euclidean distances among specimens.

3) Sexual characters. We characterized the outline of the male left paramere (part of the intromittent genital organs) in lateral view. The shape analysis was performed with the software package SHAPEv.1.3 [43] after the images of the lateral view of the paramere were converted into separate black-and-white bitmaps of the studied structures (paramere, phallobasis). SHAPE uses elliptic Fourier descriptors (EFDs) to analyze shape variation of two-dimensional outline data [44]. Four coefficients and 20 harmonics were then extracted from shape outlines and treated as shape variables (Additional file 7). Chain coding, rotation and computation of harmonics were also carried out in SHAPE. Because the male genitalia of Sericini chafers have a complex three-dimensional structure (see e.g., [45], the shape analysis of a two-dimensional projection is likely to provide a more conservative, albeit more inaccurate, estimate of the total variation. The Fourier descriptors of the specimens were analyzed with Principal Components Analyses (PCA) in XLSTAT 2007 (Addinsoft, Paris), and converted into a distance matrix to be used to compute branch lengths in PAUP*4.0b10 [42] (see below).

To determine the potential error associated with the use of the digital images for the morphometric study, we did five replicates of each image for each specimen; the images were taken by the same person, with a period of at least 2 to 24 h between replicates. The proportion of the total variation due to methodological error was quantified by dividing the trace of the pooled within-specimen covariance matrix by the trace of the total covariance matrix [46]. In addition, we did a MANOVA test to assess whether interspecific variation was significantly higher than the measurement error. MANOVA tests demonstrated that the error was significantly smaller than interspecific differences (Wilk's Lambda P ≈ 0). The covariance structure of measurement error was not significantly correlated with the covariance structure of interspecific differences. All tests of correlated evolution are based on the mean shape values from the five replicates per structure.

The morphometric signal in body and paramere shape, i.e. the resulting principal component axes (Additional file 5) explaining most of the variation in the respective traits (see Results), was investigated independently for potential overlap and significance between infraspecific and interspecific variation using MANOVA and Canonical Variate Analysis in PAST [47].

### DNA sequencing

Two mitochondrial gene regions were amplified and sequenced for the analyses: 1) cytochrome oxidase subunit 1 (*cox1*) and 2) 16S ribosomal RNA (*rrnL*), with the adjacent regions tRNA leucine (*trRNA-Leu*) and NAD dehydrogenase subunit 1 (*nad1*; mtDNA nomenclature follows [48]). The latter three gene fragments are hereafter referred as "*rrnL-nad1*". PCR and sequencing was performed using primers Pat (5'tccaatgcactaatctgccatatta) and Jerry (5'caacatttattttgattttttgg) for *cox1 *and 16SaR (5'cgcctgtttaacaaaaacat) and ND1A (5'ggtcccttacgaatttgaatatatcct) for *rrnL-nad1 *[49]. Sequencing was performed on both strands using BigDye v.2.1 (Applied Biosystems, Carslbad, US) and an ABI3700 automated sequencer in the facilities of the CIB (CSIC, Madrid). Sequences were assembled and edited using Sequencher v. 4.5 (Genecodes Corp., Ann Arbor, USA).

### Phylogenetic analysis

There was no length variation in the protein-coding genes, and variation in the ribosomal genes was minimal (see Results), so progressive alignment procedures were conducted in ClustalX 1.83 [50] under default gap opening penalty of 15 and extension penalty of 6.66. The sequences reported in this paper have been deposited in GenBank with accession numbers FJ847234-68/FJ956708-41 (Additional file 1).

To estimate the phylogenetic relationships among species of *Hymenoplia *we implemented Maximum Likelihood searches of the combined mitochondrial sequence in PhyMLv2.4.4 [51] using a GTR+I+Γ model (as selected in Modeltest 3.06, [52]), with all parameters estimated from the data. To check the stability of the results we performed additional parsimony and Bayesian analyses. Parsimony tree searches were conducted using TNT 1.0 [53], with 10 ratchet iterations, 10 cycles of tree drifting and three rounds of tree fusing for each of 200 random addition sequences, coding gaps as 5^th ^character and using five random addition sequences that included ten ratchet iterations, and three rounds of tree fusing using default settings. Bayesian analyses were conducted in MrBayes 3.12 [54] using a GTR+I+Γ model, with four partitions (each codon for *cox1 *plus *rrnL-nad1*) and estimating all parameters independently in each partition. Partition homogeneity was tested in PAUP with 100 replicates. All trees were rooted with one species of the Mediterranean genus *Paratriodonta *Baraud, found to be closely related to (but clearly outside) *Hymenoplia *[37].

Node support was assessed by the node posterior probabilities in MrBayes, and by searching 100 pseudo-replicated data sets of nonparametric bootstrapping [55] both in PhyML and TNT (generated in the latter using the parsimony ratchet).

### Comparison of the rates of evolution of the different character sets

To compare the rates of evolution of the different character systems we used as a reference tree the topology obtained with the ML search on the combined mtDNA dataset. We obtained the branch lengths of the four data sets to be compared (mitochondrial, structural characters, morphometry of the body shape, paramere outline) on this constrained topology using PAUP as follows (see e.g., [56] for a similar approach):

1) The mtDNA branch lengths, used as a reference for "neutral" change, were obtained using a GTR+I+G model with parameters estimated from the data.

2) The length of the branches of the morphological structural character matrix (Additional file 4) were determined under two different approaches: a) with parsimony [Morphology (pars)] using accelerated transformation (ACCTRAN) character-state optimization, as the number of character state changes along the branch; and b) using a mean character difference distance matrix [Morphology (dist)] derived from the morphological structural character matrix (negative branches were set to be zero). We used a distance approach in addition to the parsimony branch lengths to make the three morphological data sets directly comparable (see below).

3) The length of the branches of the body morphospace and the outline of the male parameres were obtained using a Euclidean distance matrix, obtained from the raw measurements for the body morphospace and from the normalized EFDs of the shape analysis of the parameres (Additional files 6, 7). Negative branches were set to be zero.

We compared the morphological vs. the mtDNA branch lengths at two levels (see Introduction), individual branches and patristic distances between terminals.

1) Individual branches. We did multiple correlation tests (morphology, paramere shape, body shape vs. mtDNA branches) [29,30] or ANOVA, with the amount of mtDNA change as explanatory variable, comparing it to different sets of branches (body shape, paramere shape, and morphology) grouped according to their amount of character state changes. We divided the mt branches in four (0, 0-0.001, 0.001-0.01, >0.01) or seven logarithmic categories (0, 0-0.0005, 0.0005-0.001, 0.001-0.005, 0.005-0.01, 0.01-0.05, >0.05), each having almost similar amounts of branch numbers. Since the distribution of the length of the branches of the morphological structural change was highly biased towards low values (Additional file 8) we also did the reverse ANOVA, i.e. with the length of the morphological branches as explanatory variable for the comparison with the DNA change. We divided the branches between those with no change (length = 0) and those with change (length > 0), and compared the length of the mitochondrial branches of the two groups with an ANOVA analysis. To have an overall comparison of the differences of the individual branches over the tree, we also used the K-score as measured by Ktreedist 1.0 [57]. The software provides a single metric (the K-score) for the comparison between the branch lengths and the topological differences of the two trees, with no associated significance. In our cases, the topologies of all trees were identical, so K-scores were a measure of branch length differences only. Lower values of the K-score imply less differences from the mtDNA tree, with a higher degree of similarity between the branch lengths and, consequently, a predominantly neutral mode of trait evolution. The program first calculates the scale factor that minimizes differences between trees, and then computes the branch length distance (BLD, [58]) between the scaled comparison tree and the reference tree [57]. The K-score is thus the minimum branch length distance between two trees, once one of them has been scaled. To estimate the probability that the observed K-scores were due to random processes, for each data set we obtained a null distribution of the morphological change in the same tree topology by randomizing the names of the terminals, obtaining the corresponding branch lengths in PAUP (which will now be random, with the constraint of the total amount of variation in the tree) and measuring the resulting K-score. This was computed using a Perl script (available on request), with 10,000 random replicas. To assess its significance, the observed K-score was compared to the null distribution of random K-scores [59].

2) Patristic distances. We computed the patristic distances between all terminals of the tree for the four character systems (mtDNA and three morphological data sets), and tested their association through multiple Mantel tests [60]. Significance was assessed with 10,000 permutations of the observed matrix in the program zt v1.1 [61]. Since diversifying and stabilizing selection can cause differences in the infraspecific and interspecific rates of change [e.g. [25]], we used pairwise plots of the patristic distances and least square regressions to test for possible differences in the slope of the relationship between infraspecific or interspecific morphological and mitochondrial distances (we did not use simple correlations with these data due to the non-independence of the individual measures, [17,32].

The node density effect [62-64] could affect trees with either missing data or a punctuated form of evolution, introducing artifacts through the underestimation of the measure of root to tip distances in lineages with a lower number of intermediate nodes. We tested the presence of node density effect in the trees with branch lengths estimated for each character system (mitochondrial and morphological) using the "delta" test [64], implemented online in [65]. The strength of the effect is measured with the curvilinear relationship between the number of nodes and the root to tip distance, and it is considered to be significant if the strength of the relationship is significantly greater than 0, and the degree of curvature significantly greater than unity.

## Results

### Hymenoplia phylogeny

We obtained 826 bp of the 3' end of cox1 (without length variation) and a fragment of rrnL-*tRNAleu-nad1 *varying in length between 812-816 bp (ingroup). The combined molecular data matrix included 1,651 aligned positions, with 292 parsimony informative characters. The mean uncorrected p-distance between any two sequences was 0.104% and 0.048% for the *cox1 *and *rrnL-nad1 *partitions, respectively.

**Figure 2:**
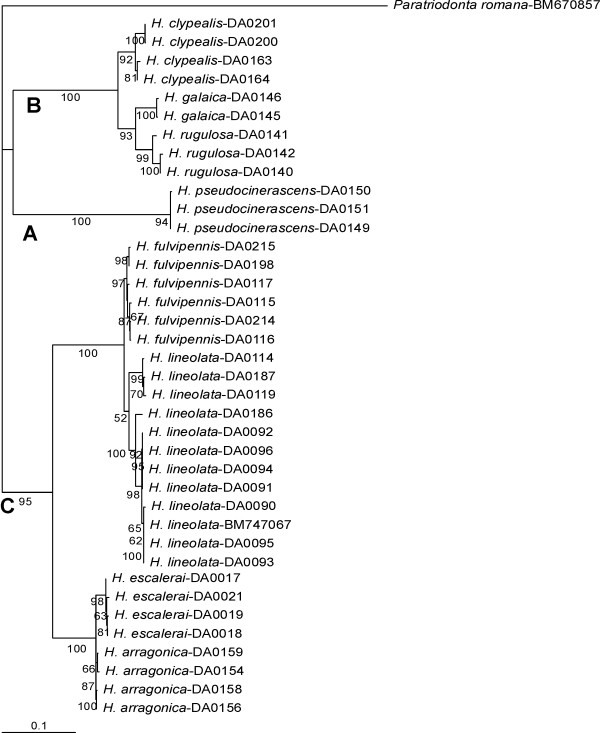
**Maximum Likelihood phylogram of the mtDNA data obtained with PhyML, including bootstrap values (only above 50% shown)**.

In all analyses *Hymenoplia *was split into three well supported clades (A, B and C) (Figure 2; see also Additional file 9). The sister group relationship between clade A and B had only low support. Within the clades A-C, all morphologically defined species were monophyletic and well supported, with the only exception of *H. lineolata *(Figure 2), which had low support in the ML analysis and was paraphyletic in the MrBayes and parsimony analyses due to the inclusion of *H. fulvipennis *(Additional file 9).

### Morphological traits

Except for structural morphology, the variation of body and paramere shape had no clear clustering according to the traditionally recognized species, with considerable overlap between them (Additional file 5). A box test (asymptotic approximation to χ^2 ^and Fischer's F) with the measurement of the total body length was not significant (p = 0.3), revealing equal intra-class variance, i.e. the amount of variation within each species is similar. On the contrary, the Wilks' lambda test for the body length was significant (p: < 0.0001), detecting significant differences in the mean body size for at least one species. The Fisher distances for the species classes were significant for about half of the inter-species comparisons, and highly negatively correlated with their significance, as revealed by a mantel test; in other words, large differences between species had the tendency to be also significant. Most of the variation of body morphospace was represented by PC axis 1 (92.7%), with all measurements being strongly correlated with it (r>0.9).

For paramere shape, 95% of the variation was represented by PC axis 1-22. However, and despite the apparent overlap (Additional file 5), differences between the scores of the species in the PC axes corresponding to 95% of total trait variation were highly significant, as measured with MANOVA (paramere shape, Wilks' lambda_95%_: 4.37E-09; p: 2.028E-20; body shape, Wilks' lambda_95%_: 0.1994; p: 1.135E-05). The same was valid for the axes representing only 75% (i.e. PC axis 1-10) of shape variation in parameres (Wilks' lambda_75%_:0.0005526; p: 4.15E-16). The Canonical Variate Analysis applied to these PC axes shows a better species discrimination for paramere shape than for body shape (Additional file 10).

### Comparison of molecular and morphological rates of evolution

For the comparison of the rates of evolution of the different character systems we used the topology of the tree obtained from ML search with the combined mtDNA (Figure 2). Branch lengths for the morphological rates of evolution were obtained in PAUP using this topology as a constraint (see Methods). None of the trees showed signs of the presence of node density effects, as measured with the "delta" test [64].

#### 1) Comparison of individual branches

**Figure 3:**
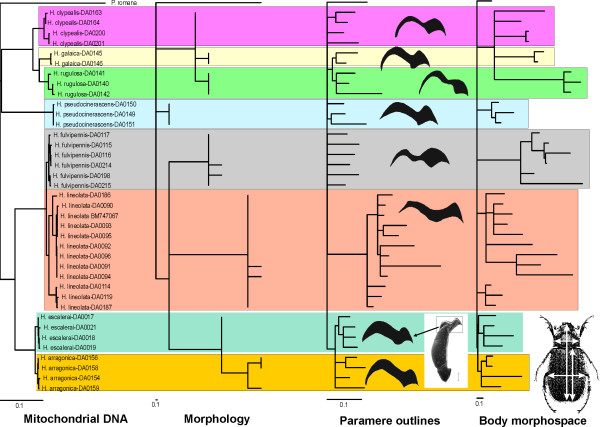
**Comparison of ML branch lengths obtained with the different character systems, optimized in the topology obtained with the mtDNA (see Figure 2)**. For the structural morphology only the parsimony reconstruction is shown (see text).

The length of the branches reflecting mtDNA change and both measures of morphospace (male parameres and general body shape; Figure 3) had approximate normal distributions (Additional file 8), and we used correlations (Pearson correlation) to estimate their relationship. All three correlations were very weak (r<0.1), and not significant (p > 0.05) (Table 1).

**Table 1:** Correlation of individual branch lengths (Pearson r) and patristic distance matrices (Mantel test) between the trees resulting from optimizing the different character systems on the ML tree topology obtained with mtDNA sequences (significant values in bold).

	**Individual branch lengths**		**Patristic distance matrices**	
	**r**	**p**	**r**	**p**
**mtDNA/Paramere outline**	0.059	0.62	0.254	**< 0.005**
**mtDNA/Body morphospace**	-0.036	0.76	0.059	0.27
**mtDNA/Morphology (pars)**	0.681	**< 0.0001**	0.620	**< 0.0001**
**mtDNA/Morphology (dist)**	0.717	**< 0.0001**	0.630	**< 0.0001**

The branches with less mtDNA change were also significantly shorter for the structural morphological characters, as measured with both ANOVAs (using four or seven categories of mtDNA change, p_4Cat _= 0.015 and p_7Cat _= 0.0003 respectively). This was also the case for the reverse comparison, i.e. the branches with no morphological change were also significantly shorter for the mtDNA change (p < 0.0005, Table 2). On the contrary, there were no significant differences in the length of the morphospace or paramere outline branches between the four and seven categories of mtDNA change (Table 2). When the branches with morphological change were subdivided in three categories (length = 0, length = 1, length > 1) the results were similar (Table 2), with significantly less mtDNA change in the branches with less morphological character state changes, but no relationship with the other two character systems. The number of branches with more than two morphological changes was too low to allow for further subdivisions of the data (Table 2; Additional file 8).

**Table 2:** ANOVA of the branch lengths of the different traits associated to changes in mtDNA using 4 and 7 different categories of change (see text).

**Source of Variation**		**SS**	**df**	**MS**	**F**	**p**
***4 categories DNA***						
***mt DNA***	Between groups	17.762	3	5.921	3.721	**0.015**
vs ***Morphology***	Within groups	112.958	71	1.591		
	Total	130.720	74			
						
***mt DNA***	Between groups	0.008	3	0.003	1.998	0.122
vs ***PmO***	Within groups	0.095	71	0.001		
	Total	0.104	74			
						
***mt DNA***	Between groups	0.070	3	0.023	0.557	0.645
vs ***Body shape***	Within groups	2.974	71	0.042		
	Total	3.044	74			
						
***7 categories DNA***						
						
***mt DNA***	Between groups	39.663	6	6.611	4.937	**0.0003**
vs ***Morphology***	Within groups	91.057	68	1.339		
	Total	130.720	74			
						
***mt DNA***	Between groups	0.010	6	0.002	1.157	0.340
vs ***PmO***	Within groups	0.094	68	0.001		
	Total	0.104	74			
						
***mt DNA***	Between groups	0.114	6	0.019	0.441	0.849
vs ***Body shape***	Within groups	2.930	68	0.043		
	Total	3.044	74			
						
***2 categories Mor***	Between groups	0.013	1	0.013	14.107	**<0.0005**
***Morphology ***	Within groups	0.069	73	0.001		
***vs mt DNA***	Total	0.083	74			
						
***3 categories Mor***	Between groups	0.020	2	0.010	11.825	**<0.00005**
***Morphology ***	Within groups	0.062	72	0.001		
***vs mt DNA***	Total	0.083	74			

SS - sum of squares; df- degree of freedom; MS - mean square; F - value of F statistic; p - P-value (significant values in bold).

**Figure 4:**
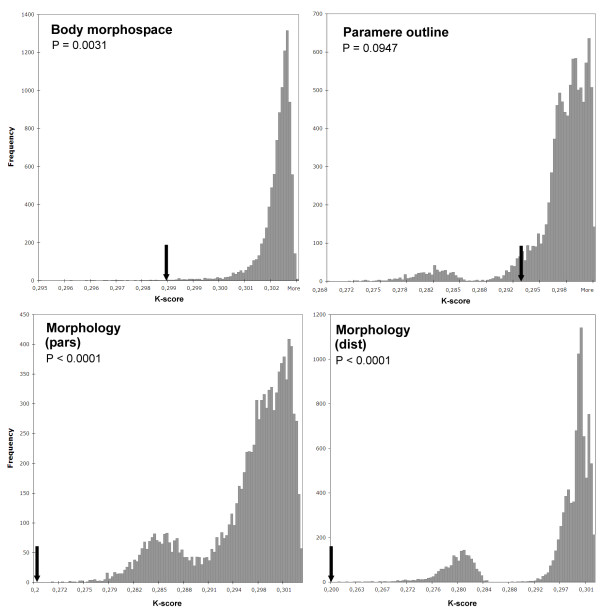
**Null distributions of the values of the K-scores between the tree with mtDNA branch lengths and the trees with a random distribution of the morphological change of the different character systems (10,000 replicas)**. The observed (i.e. non-randomized) value of the K-score is marked with an arrow. For the structural morphology characters both methods of branch length reconstruction (parsimony and distance) are shown.

We computed the null distribution of the values of the K-score between the tree with mitochondrial branch lengths and the trees with branch lengths reflecting the change in the three morphological character sets with 10,000 randomized replicas (Figure 4). Only in the comparison between the branches of the mtDNA and the paramere outline the observed value was within 95% of the null random distribution of values (Table 3; Figure 4), providing no evidence of a significant relationship between the evolutionary rates of the two character systems based on the K-score. For the body morphospace and structural morphology character systems (with both parsimony and distance branch lengths) the observed difference with the mtDNA branches was significantly lower than that expected at random, indicating a significant association between the evolutionary rates of these character systems (Table 3).

**Table 3:** K-scores between the reference tree (mitochondrial DNA) and the trees with branches reflecting morphological change of the three studied character systems (observed K-scores).

**Comparison tree**	**Scale factor**	**K-score observed**	**No. random trees with K** **lower than observed**	**Estimated p**
**Body morphospace**	0.0203	0.2972	31	0.003
**Paramere outline**	0.1786	0.2929	947	0.095
**Morphology (pars)**	0.0179	0.2116	0	<0.0001
**Morphology (dist)**	0.3812	0.2007	0	<0.0001

For the structural morphology, we calculated tree similarity for both optimisation methods, parsimony (pars) and distance (dist). Significance was tested through the use of a null distribution of 10,000 randomized trees (see Methods and Figure 4).

#### 2) Comparison of patristic distances

The Mantel tests measuring the correlation between the distance matrices of the mtDNA and structural morphology (with both parsimony and distance branch lengths) and between mtDNA and paramere outline were highly significant. On the contrary, the correlation between the mtDNA and the body morphospace matrices was not significant (Table 1).

**Figure 5:**
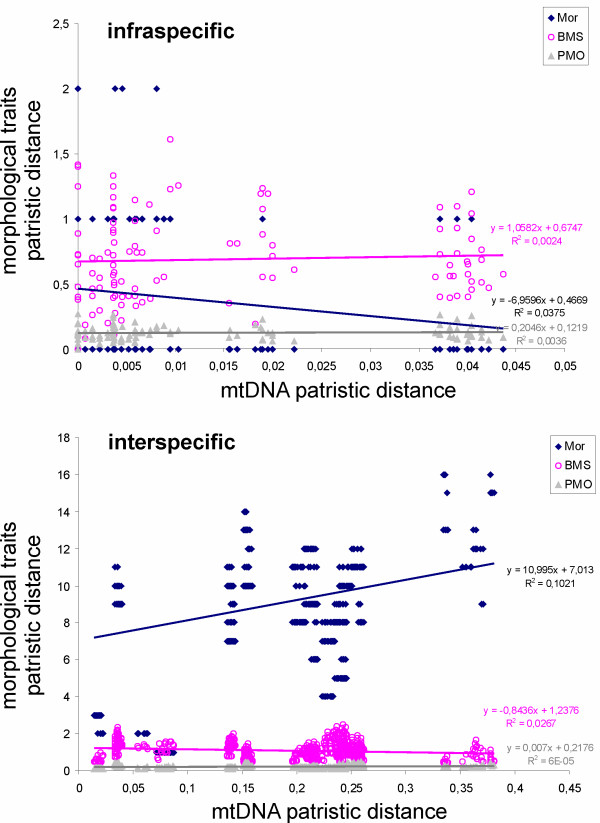
**Pairwise plots of intra- (above) and interspecific (below) patristic distances for structural morphology (Mor), body morphospace (BMS), the paramere shape (PMO) vs. mtDNA distances showing for each comparison the slopes of the regression including their equation and their coefficient of determination (R^2^)**.

The pairwise plots of intraspecific patristic distances of the body morphospace and the paramere shape vs. mitochondrial distances show no significant relationship, with slopes of the regression of shape distance vs. sequence divergent not different from zero [17]. The slope of the plot of the morphological vs. mtDNA distances was significantly negative, although with a very low correlation (R^2 ^= 0.04; p < 0.05; slope -7.0, 95% confidence interval [-13.8, -0.1]; Figure 5). For interspecific distances, the slope of the plot of the paramere outline vs. mtDNA was not significantly different from zero, but the body morphospace had a negative, and the morphology a positive slope (body morphospace, R^2 ^= 0.03; p < 0,0001; slope 0.8, 95% confidence interval [-1.2, -0.4]; morphology, R2 = 0.1; p < 0.0001; slope 11.0. 95% confidence interval [8.4, 13.51]; Figure 5).

## Discussion

In this work we exemplified the use of a novel approach to compare rates of evolution of different character systems with the study of speciation modes in a clade of Iberian Sericini chafers of the genus *Hymenoplia*. There are many factors known to influence character change and speciation, but the usual approach is to study their effect in isolation to increase the explanatory power of the statistical tests, avoiding being swamped by uncontrollable variation [10,66-68]. The simultaneous consideration of alternative mechanisms is still uncommon [14,23,69], and there is still a lack of standard approaches to compare different sources of information in phylogenetic studies.

We obtained three sets of measures of different character systems potentially subjected to different selection pressures, and tested the basic assumption that evolutionary rates should be proportional to time (i.e. "neutral") when there is no such selective pressure, but decoupled from this neutral rate when there is directional or stabilising selection acting on them [1,17,18,23]. The potential relationship between morphological and molecular evolutionary rates has been contentious, with successive re-examinations of the question in the light of new methodological approaches [30,32,33]. There are two main differences between ours and previous studies: 1) we assume that any possible correlation between the mtDNA and morphological rates is due to their independent correlation to time [17,31]; and 2) we do not aim to establish a general correlation between "molecular" and "morphological" rates, but try to determine whether different morphological character systems evolve at a neutral rate proportional to time or, on the contrary, are decoupled from the rates of mtDNA change. Thus, other than indirect effects related to population size or general pleiotropic factors [32,33], there should be no functional relationship between our measure of molecular evolution, based on mitochondrial genes, and any of the phenotypic traits considered. As we do not compare evolutionary rates among different phylogenies, we only need to establish a relative measure of the rates of different morphological character systems in the same phylogeny, minimizing problems associated with high type I errors or lack of statistical power, which are likely to be major drawbacks [33]. The fact that for almost all type of tests we find both significant and not significant results (Table 4) suggests that our methods are powerful enough to detect a relationship, but with type I errors low enough to avoid generalized false positives.

**Table 4:** Overview over the main results: association of the change in the different morphological traits with the mtDNA rates of change: ++strongly positive, + positive, n.s. non-significant.

	**Individual branches**			**Patristic distances**
	**Pearson**	**ANOVA**	**K-score**	**Mantel**

**Morphology (pars)**	++	+	++	++
**Morphology (dist)**	++	+	++	++
**Body shape**	n.s.	n.s.	+	n.s.
**Paramere shape**	n.s.	n.s.	n.s.	+

Of the three tested character systems, the evolutionary rate of "structural morphology" was in all analyses proportional to that of the mtDNA (irrespective of the method used to reconstruct the branch lengths), and thus it could be assumed that structural morphological characters evolved at rates proportional to time. Structural characters are used regularly to separate morphospecies for classification purposes, and also for phylogenetic reconstruction. For most of the characters used here (Additional file 3) there is no evidence of their biological function (e.g. small differences in the density of the pubescence or the punctuation, surface integument, color), and thus how they could drive speciation through selection. The only putative "key innovation", the tarsal claws bearing a ventral membranous fringe (Additional file 3, 4), could be highly adaptive to feeding on grasses, but is not relevant here being a synapomorphy of *Hymenoplia + Hymenochelus *[15].

On the contrary, for both body shape and size and paramere outline some of the analyses showed a decoupling of the evolutionary rates with respect to that of the mtDNA. In the case of the body shape and size, the amount of change in the individual branches of the tree was more similar to that of the mtDNA than expected by chance, as shown with the null random distribution of the K-scores. However, when the accumulation of change over the tree was compared through the patristic distances, there was no correlation between the amount of change of the mtDNA and that of the body morphospace. This suggests that when measuring individual branches the deviation from the mtDNA rate is small enough not to be significant with the tests we used (ANOVA comparisons and the null distribution of the K-scores), but when measured over the whole tree with patristic distances the accumulation of changes in the body morphospace is decoupled from the amount of mtDNA change. The lack of correlation between body shape and size and mtDNA may be interpreted as the result of homoplasy, producing "saturation" in the signal due to the independent development of similar shapes and sizes in different lineages of the tree [17,23]. This could be due to stabilizing selection favoring certain shapes and sizes (although weak enough not to be detectable when comparing individual branches, see above), or just to the existence of a limited range of possible body sizes and shapes for the whole lineage [28]. In other words, there could be a random drift or fluctuating random selection at small temporal scales, detectable with the comparison of individual branches, but over longer periods measured with the patristic distances the limited morphospace (due to stabilizing selection, functional constraints or "saturation" of the morphospace, see [17]) would cause the individual trajectories to converge and lose their proportionality to mtDNA change [28]. This is quite consistent with the dominance of the PC axis 1 for the morphospace (explaining 92% of variation) and that the discriminant analysis of the total body length revealed that general body size has some significant but not the only contribution to the variation of morphospace data.

The evolution of the shape of the paramere seems to be complementary to that of the body morphospace: when comparing individual branches the rates are not proportional to time, as would be expected under a neutral scenario [1,70], suggesting strong changes driven by factors other than pure random drift. This directional change, together with high infraspecific variation (see below), are some of the marks of sexual selection [70]. However, the patristic distances do seem to be to some extent proportional to genetic distance: even if at shorter term changes may be directional (and possibly driven by sexual selection), the accumulated change through several cladogenetic events has no directionality, becoming more like an unconstrained random drift and thus with a general correlation with genetic distance. The potential morphospace for the genital organs should be much larger than that of the body shape and size, given the wide range of different genital shapes known in Sericini in contrast with the limited variation in body shape and size (see e.g., [71]). The overall correlation of the patristic distances with the mtDNA rates suggests an unconstrained amount of change over longer periods of time, reflecting a lack of a functional role of the shape of the paramere. The unconstrained change of structural morphological characters and (to a lesser extend) paramere outline can be directly appreciated in the plots of the interspecific variation (Additional files 9, 10). On the contrary, body morphospace show a high degree of overlap, and poor discrimination among species.

### Branch correlation and the assumption of neutral mtDNA evolution

Our conclusions are based on the assumption that the mtDNA evolves neutrally and proportionally to time. This is known not to be the general case, due to heterogeneity of rates among branches (e.g., [62,72,73]), the effect of incomplete sampling and node density [63,64,74], and in some cases active selection on the mitochondrial genome [75-79].

Our conclusions should be robust to random deviations from a clock-like behavior, as they should reduce, but not bias, the informative signal. It must be noted that our null hypothesis (i.e. neutral change) is based on a positive result, not on a negative one: we do find a significant correlation between the evolutionary rates of mtDNA and those of some character systems, a result highly relevant in itself [32,33]. We did not find evidence of a node density effect, and the possible bias introduced by incomplete sampling could be expected to affect in a parallel way all character systems when plotted on the same tree topology, and thus not to introduce any strong bias in our results [19,32,80]. Although there are numerous examples of selection on mitochondrial genes (see [79] for a recent review), it is still unknown to what extend this is a widespread phenomenon that could undermine the general assumption of neutrality, especially among closely related species with a similar biology.

### Methodological issues of evolutionary rate comparisons

We used three main statistical approaches to compare the evolutionary rate of the studied character systems (Table 4): 1 correlation and ANOVA; 2) comparison of the K-scores against a null distribution; and 3) Mantel test for the correlation of the matrices of patristic distances. The first two were measures of the comparison of individual branches of the tree, and the last one of the accumulated change over the pathway from the root to the tip of each terminal taxa (Omland 1997). Standard statistics can only be applied under certain restrictions, which were not met by most of our character systems (i.e. normality; uniformity of variance [60]; see Additional file 8). For those variables showing approximate normal distributions, correlations were all not significant (Table 1), possibly due to the heterogeneity in the distribution of the change across the branches (Additional file 8) or the limited number of branches involved [33]. It seems reasonable to assume that the length of individual branches is subjected to multiple sources of variation, and that it would be difficult that simple correlations of a limited set of characters and specimens reflect any potential relationship. The comparison of the K-scores proved to be more powerful, discriminating between character systems. The absolute values of the K-score would suggest that the optimized tree based on paramere outline was closer to the mtDNA tree (K-score = 0.293) than that optimized on the body morphospace (K-score = 0.297) (Table 3). However, the randomization procedure revealed that the tree optimized on the paramere outline was not significantly different from a random tree, since the observed K-score was among the 95% of the null random distribution of values. Although the computation of the K-score includes a scaling factor, this depends on the distribution of the total amount of variation in the individual branches, so the final K-scores cannot be considered a non-dimensional number and thus comparable across character systems with different scale of change [57]. It is thus necessary to associate the K-scores to a null distribution in order to obtain meaningful results in this comparative framework. For some trees their null distribution appeared clearly bimodal (Figure 4). This suggests the presence of two discrete "stable states" within the tree, determined by the presence of two long branches: when the random tree placed these two branches together, the overall K-score decreased in a marked step, creating a bimodal distribution. The effect of long branches in originating bimodal (or trimodal) null distributions can be confirmed by simulations of trees (J. Castresana and V. Soria-Carrasco, pers. com. 2009).

### Trait change and hierarchical levels of the tree

A surprising result of our study was the amount of variation in the morphometric measures within what is usually recognized as a unique morphospecies, despite the reduced mtDNA distances (Figure 3; Additional files 5, 10). There was a perfect correspondence between morphologically recognized nominal species and mtDNA clusters of the sampled specimens (Figure 2), but some individuals within these clusters had relatively long branches for both the paramere outline and the body morphospace (Figure 3). As already noted, the difficult taxonomy of the group, and the limited amount of available data and specimens, makes difficult the rigorous assessment of the partition of the measured variation among and within species. We plotted the morphological vs. mitochondrial inter- and infraspecific distances as an exploratory tool [17]), and although the results have to be taken cautiously (due to the non-independence of the distances), they may provide useful insights into the data. The most striking difference was in the structural morphological characters, with a strong positive relationship with mtDNA distances at the interspecies level, but slightly negative (although significant) at the intraspecific level, possibly reflecting the choice of diagnostic characters of taxonomic use. It is also interesting to note the slightly (but significant) negative slope of the relationship between body morphospace and mtDNA distances, possibly reflecting the "saturation" of this character system ([17], see above) (Figure 5; Additional file 9, 10).

In any case, our basic approach does not require the delimitation of species, since it does not include species-counts or a different treatment of within- and between-species variation. It is thus not affected by potential taxonomic artefacts due to the difficult recognizability and complex taxonomy of the group [39,40], and does not depend on the partition the morphological variation (e.g., [25]).

To compare the rates of change of the character systems over the tree we used statistics giving a global measure of difference, usually based on multiple pairwise comparisons (K-scores and Mantel test on patristic distance matrices). We did not study in detail the distribution of the change in different parts of the tree, such as e.g. between sister species, or between internal and terminal branches. The examination of the branch lengths in Figure 3 suggests some interesting patterns, such as the strong divergence between the syntopically co-occurring *H. lineolata *and *H. fulvipennis*, or the frequency of deep branches with length = 0 in the paramere outline. However, the number of taxa included was insufficient to draw any conclusion from these observations, which would require more comprehensive data to be tested [33].

## Conclusion

In this work we outline an approach to compare evolutionary rates of different character systems in a clade of *Hymenoplia *chafers with the aim to study their speciation mechanisms. Although based on indirect evidence, we were able to obtain insightful conclusions on the prevalent mode of speciation, which could be the base for subsequent, more detailed ecological or biological studies. Thus, it seems likely that changes of the male genitalia are shaped by sexual selection, and that body morphospace is subject to either functional or developmental constraints with signs of stabilizing selection that cannot be unambiguously associated with speciation (Table 4). On the contrary, more structural morphological characters, including those used for species delimitation and recognition (other than male genitalia), seem to evolve at a rate proportional to time and are thus not related to speciation. These conclusions would be supported by the apparently widely occurring sympatry of the *Hymenoplia *species (Figure 1), although the taxa seem not to co-occur syntopically (see above). The possibility that sexual selection was an important factor in the diversification of the genus *Hymenoplia *is highly relevant, as much of recent biological diversity of scarabs in the Mediterranean region is attributed to vicariance only (e.g., [81,82]). The approach presented here has the additional advantage of not being dependent on species definition, and could be of potential use for comparative studies not only related to speciation, but to any question requiring the comparison of the amount of change of different character systems over a phylogenetic tree.

## Authors' contributions

DA collected the samples and performed the lab work, dissections, photography and morphometric analyses. DA and RI performed the statistical and phylogenetic comparative analyses of the data and contributed equally on the different drafts of the manuscript; figures and tables were done by DA. Both authors read and approved the final manuscript.

## Supplementary Material

Additional file 1 **Genbank accession numbers and collection data of the specimens used in the study**. Genbank accession numbers. Click here for file

Additional file 2 **Illustration of features representing the structural morphology character system: anterior body portion (head and pronotum) showing cuticular integument including pilosity, surface structure and punctation (A, B); head (C), interior lobe of protarsal claws (D, E), ventroapical spur of metatibia (F, G). A, E- *Hymenoplia escalerai*; B- *H. fulvipennis*; C- *H. clypealis*; D, G- *H. lineolata*; F- *H. arragonica *(not to scale)**. Characters used for analysis of structural morphology. Click here for file

Additional file 3 **Morphological characters including their different discrete character states used in the analysis**. Character definitions/circumscriptions (structural morphology). Click here for file

Additional file 4 **Morphological character matrix**. Morphological character matrix. Click here for file

Additional file 5 **Plots of the axes 1-3 of the principal component analyses of the variation of paramere shape and body shape (above and medium) as well of the two dimensions revealed from multidimensional scaling of structural morphological distance data (below)**. Results of the morphometric analysis. Click here for file

Additional file 6 **Morphological measurements of body distances, with the raw data in millimetres (LB- maximum total body length, PW- maximum pronotal width, EW - maximum elytral width, EL -maximum elytral length)**. Measurements of body distances. Click here for file

Additional file 7 **Raw data of paramere outline shape: normalized Elliptic Fourier Descriptors (EDFs; n = 80 [separated by slash])**. Normalized Elliptic Fourier Descriptors. Click here for file

Additional file 8 **Frequency of branch length distribution of the different traits: Body-morphospace (above left), morphology (pars, above right), paramere outline shape (below left), mtDNA (below right)**. Results of the analysis of trait change. Click here for file

Additional file 9 **Parsimony (50% majority rule consensus) and consensus phylogram obtained with MrBayes (bootstrap values above 50% and posterior probability are shown below branches)**. Results of tree searches. Click here for file

Additional file 10 **Plots of Canonical Variate Analysis showing the species-specific divergence in the quantitative morphological traits (body shape and paramere shape; the latter specified for 95%(PC axis 1-22) and 75% (PC axis 1-10) of the variation)**. Results of Canonical Variate Analysis. Click here for file
