# Supplementary material for: Inferring speciation modes in a clade of Iberian chafers from rates of morphological evolution in different character systems
Source: BMC Evol Biol. 2009 Sep 15;9:234. doi: 10.1186/1471-2148-9-234 (PMC2753572; doi:10.1186/1471-2148-9-234)
Supplement: Additional file 1 — Genbank accession numbers and collection data of the specimens used in the study. Genbank accession numbers. [file 1471-2148-9-234-S1.pdf]

Additional file 1. Genbank accession numbers and collection data of the specimens used in the study.

Species	Voucher number	Locality	Coordinates	Date	Accession no.	
					cox1	*rrnL*
Hymenoplia arragonica	DA0153	Spain: Andalusia, Arroyo de las Grajas (Sierra de Segura), E Embalse del Tranco, 709m	38°03'10.9''N, 02°48'48.9''W	24.v.2006	FJ847234	FJ956708
	DA0154				FJ847235	FJ956709
	DA0158				FJ847236	FJ956710
	DA0159				FJ847237	FJ956711
H. clypealis	DA0163	Spain: Castilla la Mancha, Puerto de Niefla (Sierra del Rey), ca 40 km SW Puertollano, 902m	38°32'08.9''N, 04°23'07.7''W	22.v.2006	FJ847238	-
	DA0164				FJ847239	FJ956712
	DA0200	Spain: Castilla la Mancha, Camping 3 km N Fuencaliente (Sierra de la Garganta), 799m	38°26'49.4''N, 04°19'37.3''W	22.v.2006	FJ847240	FJ956713
	DA0201				FJ847241	FJ956714
H. escalerai	DA0017	Spain: Alicante, Puerto de Biar, Biar	-	13.v.2006	FJ847242	FJ956715
	DA0018				FJ847243	FJ956716
	DA0019	Spain: Valencia, Balsa en cuneta, Enguera	-	6.v.2006	FJ847244	FJ956717
	DA0021	Spain: Valencia, Barranco Río Grande, Quesa	-	6.v.2006	FJ847245	FJ956718
H. fulvipennis	DA0115	Spain: Andalusia, Hinojos 14.5km E of Almonte, 117m,	37°17'21.1''N, 06°22'26.9''W	16.v.2006	FJ847246	FJ956719
	DA0116				FJ847247	FJ956720
	DA0117				FJ847248	FJ956721
	DA0198	Spain: Andalusia, Camping Torre de la Higuera, Dunes (47km SE Huelva), 47m	37°00'50.3''N, 06°34'38.9''W	16.v.2006	FJ847249	FJ956722
	DA0214	Spain: Andalusia, 2 km NE of Faro El Picach de la Barre (ca 20km SE Huelva), 51m	37°08'45.5''N, 06°44'56.4''W	17.v.2006	FJ847250	FJ956723
	DA0215				FJ847251	FJ956724
H. galaica	DA0145	Spain: Extremadura: Badajoz, 2 km E La Bazana, 5 km SE Jerez , 544m	38°14'40.6''N, 06°40'58.8''W	19.v.2006	FJ847252	FJ956725
	DA0146				FJ847253	FJ956726
H. lineolata	DA0090	Spain: Andalusia, Embalse de Retortillo env., 147m	37°50'09.5''N, 05°18'44.7''W	13.v.2006	FJ847254	FJ956727
	DA0091				FJ847270	FJ956743
	DA0092				FJ847255	FJ956728
	DA0093				FJ847256	FJ956729
	DA0094				FJ847269	FJ956742
	DA0095				FJ847257	FJ956730
	DA0096				FJ847258	FJ956731
	BM747067				EF487759	EF487803
	DA0114	Spain: Andalusia, Hinojos 14,5km E of Almonte, 117m	37°17'21.1''N, 06°22'26.9''W	16.V.2006	FJ847259	FJ956732
	DA0119				FJ847260	FJ956733
	DA0186	Spain: Andalusia, 3 km W Patrás, S of Sierra de Aracena, 282m	37°48'35.9''N, 06°43'55.5''W	19.V.2006	FJ847261	FJ956734
	DA0187				FJ847262	FJ956735
H. pseudocinerascens	DA0149	Spain: Andalusia, 5 km NW Maria (A317) (Sierra de Maria), 1243m	37°43'42.2''N, 02°13'52.1''W	25.v.2006	FJ847263	FJ956736
	DA0150				FJ847264	FJ956737
	DA0151				FJ847265	FJ956738
H. rugulosa	DA0140	Spain: Extremadura, Camping La Vera (1km E Jarandilla), 647m	40°07'22.4''N, 05°38'18.4''W	21.v.2006	FJ847266	FJ956739
	DA0141				FJ847267	FJ956740
	DA0142				FJ847268	FJ956741
Paratriodonta romana	BM670857	Italy: Lazio, Castel Porziano	-	-	EF487773	EF487910
