# Supplementary material for: Inferring speciation modes in a clade of Iberian chafers from rates of morphological evolution in different character systems
Source: BMC Evol Biol. 2009 Sep 15;9:234. doi: 10.1186/1471-2148-9-234 (PMC2753572; doi:10.1186/1471-2148-9-234)
Supplement: Additional file 2 — Illustration of features representing the structural morphology character system: anterior body portion (head and pronotum) showing cuticular integument including pilosity, surface structure and punctation (A, B); head (C), interior lobe of protarsal claws (D, E), ventroapical spur of metatibia (F, G). A, E- Hymenoplia escalerai; B- H. fulvipennis; C- H. clypealis; D, G- H. lineolata; F- H. arragonica (not to scale). Characters used for analysis of structural morphology. [file 1471-2148-9-234-S2.pdf]

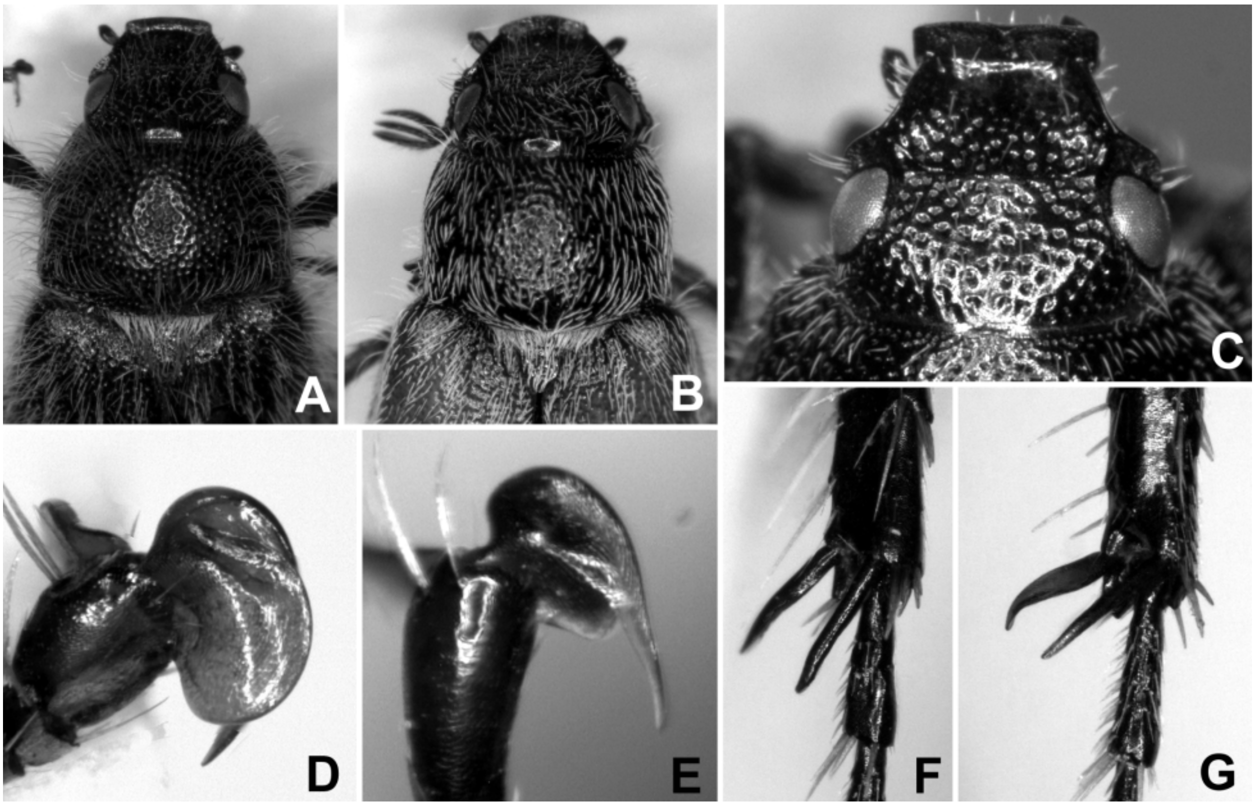

Additional file 2. Illustration of features representing the structural morphology character system: anterior body portion (head and pronotum) showing cuticular integument including pilosity, surface structure and punctation (A, B); head (C), interior lobe of protarsal claws (D, E), ventroapical spur of metatibia (F, G). A, E- *Hymenoplia escalerai*; B- *H. fulvipennis*; C- *H. clypealis*; D, G- *H. lineolata*; F- *H. arragonica* (not to scale).
