# Supplementary material for: Inferring speciation modes in a clade of Iberian chafers from rates of morphological evolution in different character systems
Source: BMC Evol Biol. 2009 Sep 15;9:234. doi: 10.1186/1471-2148-9-234 (PMC2753572; doi:10.1186/1471-2148-9-234)
Supplement: Additional file 3 — Morphological characters including their different discrete character states used in the analysis. Character definitions/circumscriptions (structural morphology). [file 1471-2148-9-234-S3.pdf]

Additional file 3. Morphological characters including their different discrete character states used in the analysis.

1. *Lateral margins of clypeus*: (0) convex; (1) concave (Suppl. Fig. 3C).
2. *Dorsal margin of labrum*: (0) uniformly rounded with the lateral margin of clypeus producing a gently rounded anterior angle; (1) produced, separated from the lateral margin of clypeus by a blunt anterior angle (Suppl. Fig. 3C).
3. *Dorsal and ventral row of galeal teeth*: (0) on the same level; (1) dorsal row basally, ventral row distally displaced.
4. *Base of pronotum (between the posterior angles)*: (0) as wide as the elytra at base (measured over humerus; Suppl. Fig. 3A); (1) distinctly narrower than the elytra at base (Suppl. Fig. 3B).
5. *Erect setae on pronotum*: (0) directed posteriorly; (1) directed on anterior portion anteriorly.
6. *Pilosity of pronotum*: (0) double (with short and long setae; Suppl. Fig. 3A); (1) simple (all setae of the same length; Suppl. Fig. 3B).
7. *Edge of intersegmental membrane at the basis of pronotum medially (ventral view)*: (0) present; (1) absent.
8. *Intervals of elytra*: (0) identical (even and odd ones); (1) odd more elevated than even ones.
9. *Color of elytra*: (0) yellowish; (1) reddish-brown; (2) black; (3) anteriorly blackish and posteriorly reddish.
10. *Long erect setae on elytra*: (0) sparse all over; (1) distinctly more dense on even intervals; (2) dense on all intervals; (3) lacking; (4) slightly more dense on even intervals.
11. *Punctures on elytra*: (0) simple, well separated; (1) touching almost each other.
12. *Epipleural keel at base of elytra*: (0) present (distinct along the entire elytra); (1) completely lacking; (2) at basal quarter present.
13. *Surface of elytra beyond shagrination*: (0) shiny; (1) dull.
14. *Tarsal claws ventrally*: (0) without membranous fringe; (1) with membranous fringe.
15. *Claws of median tarsus in male*: (0) without submedian supplemental triangular dilatation; (1) with submedian supplemental triangular dilatation.
16. *Protarsomere V (in dorsal view)*: (0) three times as long as wide at the apex; (1) at maximum twice as long as wide at the apex.
17. *Interior lobe of protarsal claws*: (0) small (equal to one third of the lateral face of protarsomere V; Suppl. Fig. 3E); (1) large (equal to about half of the lateral face of protarsomere V); (2) very large (equal to almost the entire lateral face of protarsomere V; Suppl. Fig. 3D).
18. *Ventroapical spur of metatibia*: (0) straight (Suppl. Fig. 3F); (1) strongly curved in posterior half (Suppl. Fig. 3G).
19. *Parameres*: (0) symmetrical; (1) asymmetrical.
