# Supplementary material for: Inferring speciation modes in a clade of Iberian chafers from rates of morphological evolution in different character systems
Source: BMC Evol Biol. 2009 Sep 15;9:234. doi: 10.1186/1471-2148-9-234 (PMC2753572; doi:10.1186/1471-2148-9-234)
Supplement: Additional file 4 — Morphological character matrix. Morphological character matrix. [file 1471-2148-9-234-S4.pdf]

Additional file 4. Morphological character matrix.

Char. number (10)	1	111111111
Char. number	1234567890	123456789
Paratriodonta romana-BM670857	00?0001020	020000100
Hymenoplia. arragonica-DA0154	0110100122	011100001
H. arragonica-DA0156	1110100122	011100001
H. arragonica-DA0158	1110100122	011100001
H. arragonica-DA0159	1110100122	011100001
H. clypealis-DA0163	1110000121	110101201
H. clypealis-DA0164	1110000121	110101201
H. clypealis-DA0200	1110000121	110101201
H. clypealis-DA0201	1110000121	110101201
H. escalerai-DA0017	0110100122	110100001
H. escalerai-DA0018	0110100122	110100001
H. escalerai-DA0019	0110100122	110100001
H. escalerai-DA0021	0110100122	110100001
H. fulvipennis-DA0115	0111010111	110101101
H. fulvipennis-DA0116	0111010111	110101101
H. fulvipennis-DA0117	0111010111	110101101
H. fulvipennis-DA0198	0111010131	110101101
H. fulvipennis-DA0214	0111010131	110101101
H. fulvipennis-DA0215	0111010111	110101101
H. galaica-DA0145	1110000122	110101201
H. galaica-DA0146	1110000122	110101201
H. lineolata-DA0090	0010000121	010111210
H. lineolata-DA0091	0010000131	010101110
H. lineolata-DA0094	0010??0120	01010???0
H. lineolata-DA0092	0010000121	010111210
H. lineolata-DA0093	0010000121	010111210
H. lineolata-DA0095	0010000121	010111210
H. lineolata-DA0096	0010000121	010111210
H. lineolata-DA0114	0010000121	010111210
H. lineolata-DA0119	0010000121	010111210
H. lineolata-DA0186	0010000121	010111210
H. lineolata-DA0187	0010000121	010111210
H. lineolata-BM747067	0010000121	010101210
H. pseudocinerascens-DA0149	0110000022	110101101
H. pseudocinerascens-DA0150	0110000022	110101101
H. pseudocinerascens-DA0151	0110000022	110101101
H. rugulosa-DA0140	1110100121	110101201
H. rugulosa-DA0141	1110100121	110101201
H. rugulosa-DA0142	1110100121	110101201
