# Supplementary material for: Inferring speciation modes in a clade of Iberian chafers from rates of morphological evolution in different character systems
Source: BMC Evol Biol. 2009 Sep 15;9:234. doi: 10.1186/1471-2148-9-234 (PMC2753572; doi:10.1186/1471-2148-9-234)
Supplement: Additional file 5 — Plots of the axes 1-3 of the principal component analyses of the variation of paramere shape and body shape (above and medium) as well of the two dimensions revealed from multidimensional scaling of structural morphological distance data (below). Results of the morphometric analysis. [file 1471-2148-9-234-S5.pdf]

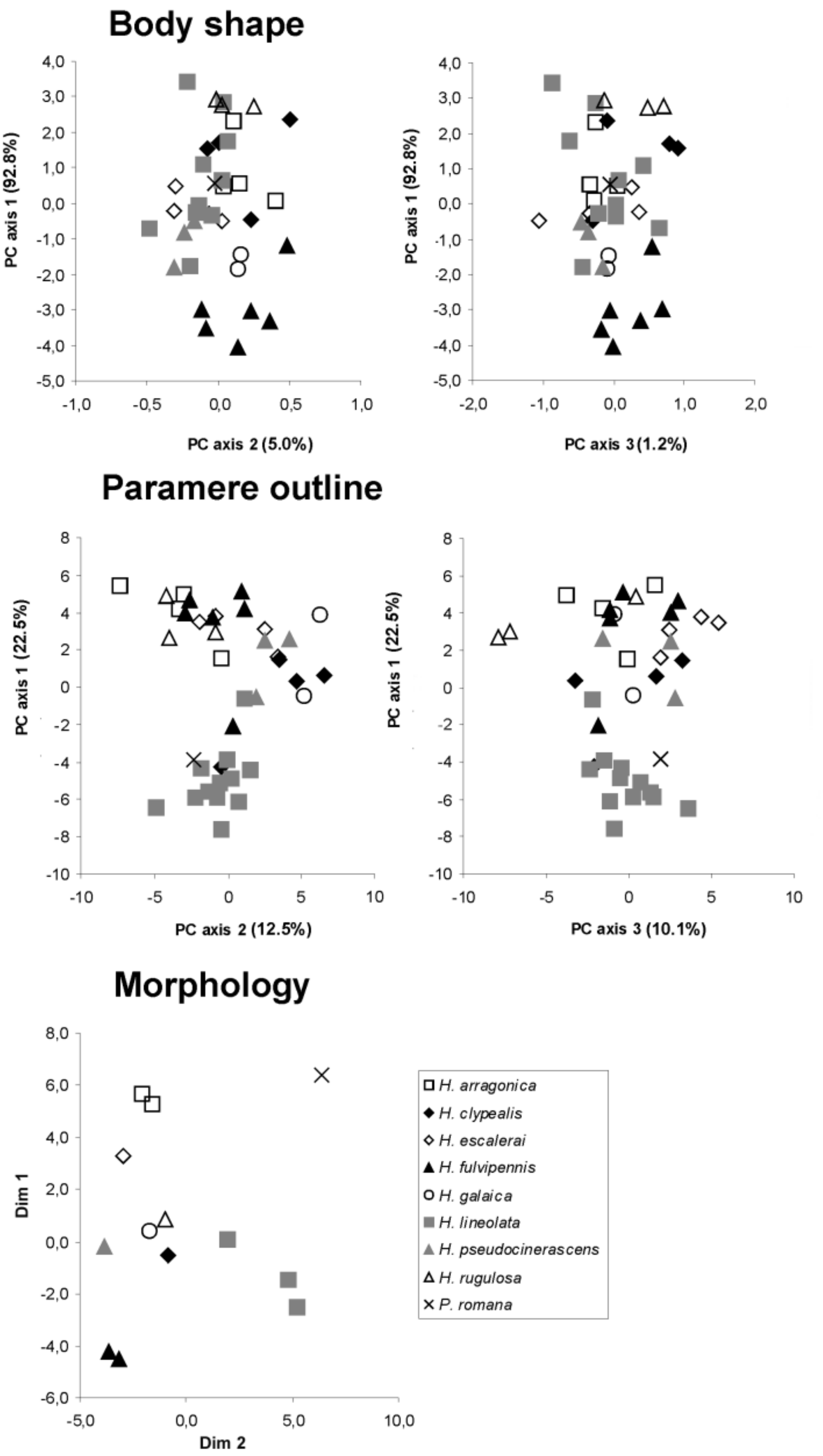

Additional file 5. Plots of the axes 1-3 of the principal component analyses of the variation of paramere shape and body shape (above and medium) as well of the two dimensions revealed from multidimensional scaling of structural morphological distance data (below).
