# Supplementary material for: Inferring speciation modes in a clade of Iberian chafers from rates of morphological evolution in different character systems
Source: BMC Evol Biol. 2009 Sep 15;9:234. doi: 10.1186/1471-2148-9-234 (PMC2753572; doi:10.1186/1471-2148-9-234)
Supplement: Additional file 6 — Morphological measurements of body distances, with the raw data in millimetres (LB- maximum total body length, PW- maximum pronotal width, EW - maximum elytral width, EL -maximum elytral length). Measurements of body distances. [file 1471-2148-9-234-S6.pdf]

Additional file 6. Morphological measurements of body distances, with the raw data in millimetres (LB- maximum total body length, PW- maximum pronotal width, EW - maximum elytral width, EL -maximum elytral length).

Specimen	LB	PW	EW	EL
Hymenoplia arragonica-DA0154	6.67	2.75	2.92	4.67
H. arragonica-DA0156	6.17	2.45	2.75	4.17
H. arragonica-DA0158	6.33	2.42	2.67	4.17
H. arragonica-DA0159	6.00	2.33	2.67	4.17
H. clypealis-DA0163	6.67	2.67	2.92	4.83
H. clypealis-DA0164	5.83	2.33	2.50	4.08
H. clypealis-DA0200	7.00	2.50	2.67	4.50
H. clypealis-DA0201	7.00	2.50	2.75	4.50
H. escalerai-DA0017	6.50	2.42	2.67	4.00
H. escalerai-DA0018	5.58	2.42	2.67	3.83
H. escalerai-DA0019	6.33	2.33	2.50	3.92
H. escalerai-DA0021	6.00	2.33	2.67	3.83
H. fulvipennis-DA0115	5.33	1.75	2.08	3.42
H. fulvipennis-DA0116	5.00	1.75	2.00	3.17
H. fulvipennis-DA0117	5.67	1.83	2.08	3.33
H. fulvipennis-DA0198	6.00	2.00	2.42	3.92
H. fulvipennis-DA0214	5.25	1.88	2.17	3.42
H. fulvipennis-DA0215	5.17	1.83	2.17	3.08
H. galaica-DA0145	5.58	2.08	2.33	3.67
H. galaica-DA0146	5.67	2.17	2.33	3.83
H. lineolata-DA0090	6.33	2.50	2.58	4.33
H. lineolata-DA0091	6.00	2.42	2.50	4.00
H. lineolata-DA0092	5.50	2.22	2.33	3.58
H. lineolata-DA0093	6.17	2.42	2.50	4.08
H. lineolata-DA0094	6.83	2.83	3.00	4.75
H. lineolata-DA0095	6.33	2.75	2.83	4.55
H. lineolata-DA0096	6.75	3.08	3.08	4.83
H. lineolata-DA0114	6.08	2.33	2.50	4.00
H. lineolata-DA0119	6.33	2.28	2.33	3.83
H. lineolata-DA0186	6.67	2.50	2.67	4.33
H. lineolata-DA0187	6.00	2.42	2.50	4.00
H. lineolata-BM747067	6.67	2.50	2.67	4.33
H. pseudocinerascens-DA0149	5.67	2.17	2.33	3.50
H. pseudocinerascens-DA0150	5.83	2.42	2.50	3.92
H. pseudocinerascens-DA0151	5.83	2.33	2.50	3.75
H. rugulosa-DA0140	7.25	2.67	2.92	4.75
H. rugulosa-DA0141	7.08	2.67	2.92	4.83
H. rugulosa-DA0142	6.92	2.83	3.00	4.75
Paratriodonta romana-BM670857	6.30	2.47	2.65	4.20
