# Supplementary material for: Inferring speciation modes in a clade of Iberian chafers from rates of morphological evolution in different character systems
Source: BMC Evol Biol. 2009 Sep 15;9:234. doi: 10.1186/1471-2148-9-234 (PMC2753572; doi:10.1186/1471-2148-9-234)
Supplement: Additional file 7 — Raw data of paramere outline shape: normalized Elliptic Fourier Descriptors (EDFs; n = 80 [separated by slash]). Normalized Elliptic Fourier Descriptors. [file 1471-2148-9-234-S7.pdf]

Additional file 7. Raw data of paramere outline shape: normalized Elliptic Fourier Descriptors (EDFs; n=80 [separated by slash]).

***H. arragonica*-DA0154:** 1.00E+00/7.65E-18/-7.47E-17/3.14E-01/4.31E-03/-9.19E-03/-1.10E-01/5.03E-
02/6.47E-02/3.81E-03/7.81E-02/6.92E-02/2.86E-02/-1.71E-02/-1.06E-01/-4.24E-02/4.99E-03/-8.26E-
05/-2.91E-02/-1.53E-02/4.51E-04/-1.58E-02/-2.10E-02/-1.57E-02/3.93E-03/-5.58E-03/-1.66E-
03/3.83E-02/1.98E-03/-1.28E-02/2.52E-03/9.82E-03/8.70E-04/-5.26E-03/-8.68E-03/1.22E-02/-1.45E-
03/-3.75E-03/-2.27E-03/-6.07E-03/-3.38E-03/1.87E-03/-1.13E-03/4.34E-03/-2.06E-05/-3.26E-
03/1.89E-03/3.24E-03/-2.41E-04/-1.67E-03/-1.93E-03/7.68E-03/1.98E-03/-4.62E-03/4.53E-03/-1.22E-
05/-1.97E-03/-7.73E-04/-2.03E-03/1.50E-03/-8.94E-04/-6.00E-043.41E-03 2.51E-03 -1.09E-03 1.31E-
05 -4.69E-04 1.37E-03 2.67E-04 -1.20E-03 2.29E-03 3.61E-04 -5.98E-04 -9.30E-04 4.54E-04 -1.41E-
03 -3.40E-04 1.64E-05 2.60E-03 6.97E-04

***H. arragonica*-DA0156:** 1.00E+00/-6.54E-17/1.90E-16/3.19E-01/3.43E-02/4.16E-03/-1.84E-01/3.92E-
02/4.57E-02/2.43E-02/1.45E-01/1.09E-01/2.83E-02/-1.41E-02/-4.48E-02/-4.12E-03/1.87E-02/8.21E-
03/-2.66E-02/1.62E-02/3.11E-03/-2.31E-02/-1.83E-02/-3.38E-02/5.87E-03/4.15E-03/-2.80E-02/-5.08E-
03/-5.88E-03/-1.02E-02/-1.02E-02/-2.78E-03/-2.50E-03/2.60E-03/2.41E-03/1.41E-02/1.80E-03/-8.58E-
03/1.00E-02/4.77E-03/-2.21E-03/-3.18E-03/6.77E-04/1.24E-02/1.76E-03/-7.60E-03/3.36E-03/5.08E-
03/-4.66E-03/-1.38E-03/-7.08E-03/-2.61E-03/-1.06E-03/-2.54E-03/-1.87E-03/3.37E-04/-4.63E-
03/2.68E-03/-3.01E-03/1.43E-03/6.43E-04/-4.26E-044.20E-03 2.37E-03 -2.12E-03 -1.65E-03 2.25E-03
3.19E-04 7.67E-04 -2.40E-03 3.16E-03 3.44E-03 -2.40E-03 -1.68E-03 1.54E-03 -1.24E-03 -3.94E-04
1.07E-04 -3.95E-04 1.11E-03

***H. arragonica*-DA0158:** 1.00E+00/1.05E-17/-5.73E-17/2.91E-01/6.26E-02/4.22E-03/-2.44E-01/3.01E-
02/3.67E-02/1.25E-02/1.50E-01/9.41E-02/3.36E-02/6.62E-04/-3.66E-02/-4.79E-03/1.21E-02/-1.88E-
03/-2.50E-02/3.14E-02/3.27E-03/-7.96E-03/-2.89E-02/-3.39E-02/8.56E-03/-1.47E-02/-2.02E-02/-
4.17E-03/-5.02E-04/-5.10E-03/-1.83E-02/-1.37E-02/-4.37E-04/-6.06E-03/-7.02E-03/1.45E-02/-2.96E-
03/-6.12E-03/8.74E-03/3.37E-03/2.69E-03/-4.40E-03/-1.65E-03/1.32E-02/-2.15E-03/-3.03E-03/5.71E-
03/6.00E-03/1.90E-03/-2.56E-03/-5.39E-03/3.90E-03/-8.12E-04/-2.48E-03/-5.80E-04/-2.49E-03/4.98E-
04/-1.52E-04/-7.31E-03/8.83E-05/-1.21E-03/-1.48E-031.52E-03 2.65E-03 -1.98E-03 6.17E-04 -3.48E-
04 9.87E-04 -9.32E-04 -2.38E-03 2.21E-03 5.35E-03 -1.69E-03 -6.54E-04 2.31E-03 1.33E-03 2.66E-04
-1.01E-03 2.77E-03 1.86E-03

***H. arragonica*-DA0159:** 1.00E+00/3.14E-17/-8.64E-17/2.68E-01/3.86E-02/8.84E-03/-2.54E-01/5.22E-
02/4.48E-02/8.35E-04/1.65E-01/1.07E-01/3.54E-02/2.78E-03/-4.89E-02/-6.08E-03/1.41E-02/-6.09E-
03/-3.57E-02/1.79E-02/-8.35E-03/-1.93E-02/-3.35E-02/-3.52E-02/8.23E-03/-7.55E-03/-1.33E-02/-
9.81E-03/-2.35E-03/-9.42E-03/-7.51E-03/-1.91E-03/-9.48E-04/-1.43E-03/3.99E-04/2.69E-02/-1.62E-
03/-6.66E-03/2.62E-03/6.11E-03/1.76E-03/-5.39E-03/-1.55E-03/1.34E-02/9.43E-04/-6.34E-03/5.20E-
03/1.09E-03/-6.70E-04/2.35E-05/-2.20E-03/1.91E-03/-4.24E-03/-2.48E-03/-1.21E-04/-2.51E-03/-
3.86E-03/2.08E-03/-2.30E-03/2.40E-03/-4.22E-05/-1.22E-034.33E-03 2.09E-03 -2.40E-03 3.94E-04 -
8.57E-05 6.92E-04 3.96E-04 -2.18E-03 3.82E-03 7.90E-04 -1.55E-03 -8.71E-04 2.25E-03 1.64E-04
6.62E-04 -1.09E-03 2.23E-03 -8.22E-05

***H. clypealis*-DA0163:** 1.00E+00/7.16E-18/-4.55E-17/1.99E-01/2.66E-02/1.57E-02/-2.17E-01/6.12E-02/5.43E-
02/5.64E-03/1.14E-01/8.16E-02/2.68E-02/-2.41E-03/-1.08E-01/-2.53E-02/2.31E-03/-1.83E-02/-5.01E-
02/-5.99E-03/-7.12E-03/-6.62E-03/-2.94E-02/-1.98E-02/3.92E-03/-5.56E-03/9.67E-04/2.99E-02/-
5.52E-03/-2.37E-04/-8.55E-03/1.52E-02/3.00E-03/-2.87E-03/3.72E-03/2.02E-02/-2.04E-04/-1.42E-03/-
1.04E-03/-4.42E-03/1.47E-03/-3.98E-04/-1.42E-03/4.98E-03/-3.83E-03/4.45E-05/-9.40E-04/1.87E-
03/2.64E-03/3.61E-04/-1.69E-03/1.08E-02/-1.17E-03/-2.22E-03/2.70E-03/1.21E-03/1.54E-03/5.88E-
04/2.69E-03/2.67E-03/-7.49E-04/-1.69E-034.69E-03 -4.00E-04 -1.52E-03 1.25E-03 -1.42E-03 2.27E-
03 -3.80E-04 -1.84E-03 -7.73E-04 6.42E-04 4.28E-04 -1.12E-04 -1.29E-03 2.30E-03 -3.51E-04 -1.76E-
03 2.98E-03 -4.30E-04

***H. clypealis*-DA0164:** 1.00E+00/-6.23E-18/4.27E-17/2.51E-01/-3.41E-03/5.82E-03/-1.71E-01/5.77E-02/7.00E-
02/1.21E-02/1.35E-01/1.04E-01/3.47E-02/-9.55E-03/-1.08E-01/-1.81E-02/8.62E-03/-1.31E-02/-5.11E-
02/-1.23E-02/-7.67E-03/-1.46E-02/-2.98E-02/-2.53E-02/-1.10E-03/1.10E-03/4.63E-03/2.57E-02/-
5.94E-03/-4.31E-03/2.39E-04/1.78E-02/7.70E-04/-5.44E-03/8.57E-03/2.49E-02/3.70E-03/-7.06E-03/-
1.51E-03/-2.86E-03/-2.50E-03/1.09E-03/-1.25E-03/3.89E-03/-5.61E-03/2.35E-03/1.52E-04/-9.71E-04/-
3.52E-03/3.61E-03/-1.94E-04/8.28E-03/5.32E-04/-9.06E-04/6.25E-03/1.39E-03/2.05E-03/-2.34E-
03/2.51E-03/3.15E-03/-1.33E-03/-2.86E-035.09E-03 -2.76E-03 -2.48E-03 6.24E-04 -8.30E-04 2.07E-
03 -1.35E-03 1.96E-03 2.31E-03 1.78E-03 6.39E-04 1.69E-03 -5.37E-04 1.47E-03 1.27E-03 -4.34E-04
3.69E-03 -1.32E-03

***H. clypealis*-DA0200:** 1.00E+00/2.95E-17/-6.79E-17/2.83E-01/-5.80E-03/-1.17E-02/-1.55E-01/5.95E-02/5.33E-
02/3.91E-04/1.18E-01/9.85E-02/2.78E-02/-2.10E-02/-1.03E-01/-1.90E-02/6.24E-03/-9.68E-03/-4.64E-
02/1.24E-03/9.82E-04/-7.86E-03/-2.77E-02/-1.90E-02/-4.25E-03/-2.83E-03/1.59E-03/2.76E-02/-1.12E-
02/-9.38E-03/7.07E-03/9.90E-03/-7.44E-04/-9.46E-03/7.91E-03/2.64E-02/-6.72E-04/-2.26E-03/5.70E-

03/4.89E-03/4.74E-04/8.43E-04/-6.95E-03/3.73E-03/-7.91E-04/6.51E-04/3.71E-03/-6.90E-03/-4.00E-03/3.43E-04/1.94E-05/1.92E-03/-3.36E-03/-1.66E-03/6.34E-03/2.85E-03/-1.61E-03/-2.22E-03/2.37E-03/4.21E-03/1.35E-03/-1.98E-034.88E-03 5.83E-05 -1.80E-04 6.19E-04 -1.37E-03 -3.79E-03 -1.38E-03 6.43E-04 1.97E-03 -1.29E-03 -2.14E-03 1.84E-03 -6.24E-04 -6.22E-04 -1.17E-03 1.11E-03 2.18E-03 2.77E-03

***H. clypealis*-DA0201:** 1.00E+00/-1.02E-17/8.05E-17/2.40E-01/-2.17E-02/-9.50E-03/-2.90E-02/6.15E-02/1.05E-01/-2.51E-02/1.49E-02/4.77E-02/-8.21E-03/-1.71E-02/-1.43E-01/-3.96E-02/1.55E-02/8.63E-03/-1.94E-02/1.01E-02/-5.11E-03/-1.04E-02/5.98E-03/2.00E-03/1.91E-03/-1.03E-02/6.58E-03/2.26E-02/-9.36E-03/-8.53E-03/-1.70E-02/5.13E-03/3.76E-03/1.86E-03/9.87E-04/1.41E-02/8.53E-04/-3.36E-03/-1.08E-03/-4.66E-04/-1.17E-03/1.34E-03/6.65E-04/4.58E-03/-3.05E-03/-2.77E-03/-2.34E-03/-5.83E-04/-2.62E-04/5.12E-04/4.80E-03/5.09E-03/-4.70E-04/-4.10E-03/4.70E-03/1.55E-03/-3.05E-04/-1.35E-04/3.15E-05/5.72E-03/7.30E-05/-6.54E-04-6.90E-04 -2.30E-03 1.89E-05 7.16E-04 9.90E-04 5.11E-04 -1.23E-03 -1.87E-03 3.83E-03 -1.30E-03 -1.67E-03 -1.20E-04 8.25E-04 3.14E-03 -4.04E-04 -2.04E-03 2.20E-03 -7.37E-04

***H. escalerai*-DA0017:** 1.00E+00/1.37E-17/-6.56E-17/3.05E-01/1.64E-02/-3.53E-03/-1.93E-01/4.35E-02/6.19E-02/9.75E-04/1.30E-01/7.49E-02/2.40E-02/-6.17E-03/-7.15E-02/-1.52E-03/1.35E-02/-8.75E-03/-4.61E-02/1.50E-02/-1.07E-02/-7.10E-03/-3.59E-02/-3.72E-02/3.57E-03/-5.79E-03/-1.25E-02/1.55E-02/-5.53E-03/-4.37E-03/6.64E-03/1.24E-02/3.79E-04/-2.53E-03/8.40E-03/2.42E-02/-1.40E-05/-7.75E-03/2.14E-03/5.65E-03/-2.72E-03/-7.32E-04/-4.96E-03/7.12E-03/-2.53E-03/3.75E-04/-1.35E-03/-4.19E-03/-9.45E-05/2.23E-03/-2.64E-04/4.44E-03/-1.19E-03/1.07E-04/9.82E-03/2.57E-03/1.03E-03/-6.87E-04/-1.47E-04/2.99E-03/-2.24E-03/-2.54E-033.65E-03 1.33E-03 3.49E-04 -1.95E-04 -3.14E-04 -9.39E-04 -2.31E-04 -1.84E-04 3.26E-03 -4.48E-04 -6.41E-04 8.32E-04 1.03E-03 -7.07E-04 -7.98E-04 -5.62E-04 2.02E-03 6.95E-04

***H. escalerai*-DA0018:** 1.00E+00/4.14E-18/-1.32E-17/3.34E-01/4.46E-02/5.29E-03/-1.96E-01/2.36E-02/3.80E-02/7.46E-04/1.23E-01/7.12E-02/3.58E-02/-6.86E-03/-8.35E-02/-1.91E-02/3.53E-03/-8.58E-03/-4.91E-02/2.56E-02/-7.42E-04/-7.97E-03/-2.99E-02/-2.25E-02/3.77E-03/-5.09E-03/-1.46E-02/2.07E-02/-5.86E-03/-1.37E-03/8.61E-03/9.54E-03/2.13E-03/-4.97E-03/8.68E-03/2.15E-02/-2.28E-03/-3.00E-03/7.92E-03/4.63E-03/-1.52E-05/-1.82E-03/-8.74E-03/8.74E-03/-2.79E-03/8.73E-04/9.30E-05/2.40E-04/2.41E-03/3.74E-04/-3.72E-03/2.24E-03/-3.45E-04/-3.37E-04/1.02E-02/2.59E-03/-1.67E-03/-1.55E-03/3.56E-03/-1.17E-04/-2.05E-03/-1.93E-033.00E-03 1.02E-03 -2.40E-03 -3.91E-04 -5.24E-04 -2.25E-04 2.59E-04 2.73E-04 1.91E-03 2.41E-03 9.07E-04 -5.35E-04 8.15E-05 -2.72E-03 -1.13E-03 -2.74E-06 1.51E-03 5.29E-04

***H. escalerai*-DA0019:** 1.00E+00/-1.40E-17/1.08E-17/3.47E-01/1.56E-02/-1.87E-02/-1.34E-01/6.80E-03/5.65E-02/-1.34E-03/1.16E-01/8.68E-02/2.29E-02/-2.10E-02/-3.95E-02/-1.51E-02/1.28E-02/9.78E-04/-4.89E-02/2.43E-02/5.65E-04/-1.47E-02/-2.74E-02/-2.35E-02/4.55E-03/-4.85E-04/-2.74E-02/4.57E-03/-1.66E-04/-7.84E-03/6.04E-03/1.78E-03/-3.31E-03/-5.67E-03/7.97E-03/1.41E-02/3.61E-04/-9.16E-03/1.40E-02/7.18E-03/-3.71E-03/-4.72E-03/-3.24E-03/7.36E-03/-3.73E-04/-6.26E-04/3.04E-03/7.53E-03/-2.93E-04/2.47E-03/-4.04E-03/-8.07E-04/1.83E-04/2.15E-03/3.95E-03/3.06E-03/-1.81E-03/4.48E-05/3.46E-03/-3.40E-03/-1.66E-03/-4.99E-043.53E-03 1.68E-03 -1.40E-03 -2.21E-03 2.43E-03 -5.44E-04 -5.38E-04 1.87E-04 2.27E-04 2.53E-03 2.47E-04 6.73E-05 2.02E-03 -1.14E-03 -2.72E-04 6.70E-04 1.34E-04 -6.56E-05

***H. escalerai*-DA0021:** 1.00E+00/2.39E-17/-7.73E-17/3.38E-01/2.88E-02/-1.24E-02/-1.54E-01/2.38E-02/5.07E-02/1.14E-02/1.17E-01/7.90E-02/2.93E-02/-1.78E-02/-3.78E-02/-1.27E-02/1.03E-02/3.35E-04/-3.85E-02/3.21E-02/-2.58E-03/-1.12E-02/-2.98E-02/-2.61E-02/3.70E-03/-4.39E-03/-2.51E-02/1.30E-02/-1.65E-04/-6.91E-03/5.77E-03/1.18E-03/-1.93E-03/-2.32E-03/4.84E-03/1.17E-02/5.41E-04/-7.34E-03/1.13E-02/6.55E-03/-4.81E-03/-3.51E-03/-4.89E-03/6.32E-03/-9.25E-04/-1.83E-03/-4.66E-04/2.27E-03/-2.00E-03/-6.46E-04/-5.29E-03/1.96E-03/1.30E-03/2.26E-04/5.31E-03/3.51E-03/-1.32E-03/-5.75E-04/3.22E-03/-3.04E-03/-1.44E-03/-1.14E-035.29E-03 2.11E-03 -1.73E-03 -1.58E-03 2.13E-03 -1.98E-03 -1.09E-03 7.26E-04 1.10E-03 1.54E-03 -1.58E-04 -1.06E-03 1.30E-03 -1.23E-03 -7.31E-05 9.22E-04 4.18E-04 -3.46E-04

***H. fulvipennis*-DA0115:** 1.00E+00/-3.62E-17/6.92E-17/3.62E-01/6.53E-02/-3.50E-03/-1.78E-01/-1.79E-02/4.44E-02/-1.32E-02/8.93E-02/9.63E-02/2.32E-02/-5.26E-03/-8.02E-02/-7.91E-03/4.67E-03/-6.13E-04/-2.61E-02/8.89E-03/2.34E-03/-1.68E-02/1.16E-03/-2.84E-02/2.33E-03/-4.60E-03/-1.72E-02/9.08E-03/-9.36E-04/-2.16E-03/-1.15E-02/1.61E-02/1.64E-03/-7.19E-03/-1.41E-03/2.63E-02/-1.45E-03/-1.53E-03/8.02E-03/-2.53E-03/3.64E-03/-1.34E-03/-6.66E-03/3.31E-03/-6.30E-03/-2.68E-03/1.25E-02/5.36E-03/-1.15E-03/3.31E-03/-3.51E-03/-1.22E-03/-1.03E-03/-5.28E-03/1.87E-03/7.87E-03/-2.45E-03/2.10E-03/2.65E-03/-2.60E-03/3.19E-03/-1.08E-03-2.40E-03 2.05E-03 -3.58E-03 -6.80E-04 2.39E-03 9.63E-04 -2.22E-04 5.73E-04 3.41E-03 4.58E-04 -1.69E-03 -1.03E-03 2.89E-03 -1.75E-03 -7.86E-04 -5.82E-05 5.55E-04 1.13E-03

***H. fulvipennis*-DA0116:** 1.00E+00/-7.72E-18/4.61E-17/2.25E-01/9.54E-03/-5.59E-03/-6.52E-02/9.24E-02/8.78E-02/6.86E-03/5.67E-02/7.67E-02/1.28E-02/-1.97E-03/-1.13E-01/-2.70E-02/1.98E-02/-1.18E-03/3.10E-02/-2.74E-02/2.17E-03/-1.04E-02/-2.76E-02/-1.39E-02/-1.85E-04/-5.86E-03/-2.80E-

02/3.41E-02/4.40E-03/-8.46E-03/-1.37E-03/1.16E-02/7.72E-03/-1.73E-03/9.81E-03/9.53E-03/-7.07E-03/-7.38E-03/-7.93E-03/-1.98E-03/1.25E-03/-2.16E-03/1.63E-03/7.39E-03/2.01E-05/-3.42E-03/-7.07E-04/5.72E-04/-1.24E-03/1.10E-03/3.58E-04/3.90E-03/-4.88E-04/-3.43E-03/1.01E-03/-1.32E-04/4.37E-04/-8.26E-04/1.31E-03/6.86E-03/-1.53E-03/-1.47E-035.15E-04 -1.37E-03 -7.09E-04 1.04E-03 1.14E-03 1.54E-05 4.25E-04 -1.34E-03 1.57E-03 -9.01E-04 -2.40E-04 -1.12E-05 -1.66E-04 2.38E-03 3.79E-04 -1.50E-03 9.36E-04 -2.83E-04

***H. fulvipennis*-DA0117:** 1.00E+00/8.18E-18/-1.14E-17/2.66E-01/1.74E-02/-1.16E-02/-1.59E-01/1.19E-02/5.44E-02/3.26E-03/1.39E-01/1.18E-01/3.34E-02/-3.34E-04/-6.48E-02/-7.18E-04/1.26E-02/-4.35E-03/-5.27E-02/1.67E-02/2.97E-03/-1.58E-02/-2.97E-02/-3.12E-02/5.03E-03/-3.95E-03/-1.80E-02/5.43E-03/-6.93E-03/-8.67E-03/1.08E-03/7.01E-03/-4.80E-03/-2.12E-03/1.30E-03/2.34E-02/1.72E-03/-6.43E-03/8.84E-03/1.19E-02/3.15E-04/-3.78E-03/-3.14E-04/5.71E-03/2.35E-03/-3.52E-03/3.84E-03/-4.12E-03/-2.38E-03/7.63E-04/-3.60E-03/-3.19E-03/-1.92E-03/-6.41E-04/2.30E-03/5.37E-03/-1.92E-03/4.35E-04/-1.31E-04/2.20E-03/5.08E-04/-2.42E-034.51E-03 3.86E-03 3.21E-04 -2.11E-03 3.33E-03 -1.79E-03 1.17E-03 -1.42E-03 2.82E-03 -1.69E-04 -1.76E-03 3.96E-04 1.72E-03 -2.64E-03 -1.39E-04 1.02E-04 -1.44E-03 1.28E-03

***H. fulvipennis*-DA0198:** 1.00E+00/-2.98E-17/1.09E-16/2.77E-01/6.12E-02/2.31E-02/-2.57E-01/3.69E-02/4.45E-02/9.26E-03/1.16E-01/1.03E-01/3.54E-02/6.06E-03/-5.95E-02/-1.92E-02/1.31E-02/-1.36E-02/-3.92E-02/8.29E-03/-1.73E-03/-9.82E-03/-2.03E-02/-3.28E-02/2.10E-04/-6.80E-03/-1.23E-02/1.58E-02/6.87E-04/-2.01E-03/-3.74E-03/1.28E-02/9.11E-04/-1.84E-03/-3.31E-03/2.50E-02/3.59E-03/-2.97E-03/4.71E-03/1.71E-03/1.01E-03/-3.30E-03/-3.31E-03/6.47E-03/-1.33E-03/-2.35E-03/5.86E-03/-3.07E-03/-3.24E-03/-1.05E-04/-2.41E-03/8.81E-04/7.07E-04/-9.72E-04/2.63E-03/5.08E-03/-1.00E-03/-8.88E-04/-1.41E-03/4.46E-03/6.42E-04/-1.12E-033.55E-03 -3.36E-05 -2.48E-04 -1.86E-03 2.90E-04 -1.32E-03 -7.90E-04 -4.61E-04 4.27E-03 1.32E-03 -1.27E-03 -2.80E-04 1.19E-03 -1.92E-03 1.18E-04 -1.09E-03 1.22E-03 1.67E-03

***H. fulvipennis*-DA0214:** 1.00E+00/-7.22E-21/-2.84E-17/2.78E-01/6.41E-02/5.69E-03/-2.07E-01/3.78E-02/3.92E-02/-1.04E-03/1.05E-01/9.83E-02/3.94E-02/1.09E-02/-9.01E-02/-1.09E-02/4.05E-03/-1.46E-02/-1.92E-02/8.01E-03/-1.04E-03/-1.16E-02/-1.06E-02/-4.18E-02/5.67E-03/-9.31E-03/-1.89E-02/2.42E-02/-6.70E-03/-1.00E-03/-8.72E-03/1.82E-02/3.19E-03/-6.14E-04/-2.12E-03/1.96E-02/2.43E-04/-3.51E-03/3.01E-03/-3.26E-04/-8.55E-06/-1.23E-03/1.47E-03/7.40E-03/-1.02E-03/-1.70E-03/5.34E-03/-1.87E-03/-2.46E-03/-3.00E-04/-6.49E-03/4.44E-03/-1.56E-03/1.32E-04/1.60E-03/4.46E-03/1.91E-04/-8.28E-04/-1.12E-03/2.12E-03/-3.18E-04/-1.18E-034.40E-03 2.10E-03 -1.64E-03 -7.91E-04 6.28E-04 1.95E-03 -1.28E-03 -1.95E-03 3.73E-03 1.47E-03 -8.98E-04 -2.27E-04 -6.01E-04 -2.04E-03 -4.89E-04 2.73E-04 2.21E-03 4.27E-04

***H. fulvipennis*-DA0215:** 1.00E+00/-4.37E-17/1.42E-16/2.75E-01/1.42E-02/1.72E-03/-8.54E-02/2.92E-02/6.36E-02/8.99E-03/8.86E-02/9.81E-02/2.77E-02/4.17E-03/-8.57E-02/-2.21E-02/2.09E-02/2.36E-03/-1.25E-02/-4.94E-03/6.44E-03/-8.32E-03/-1.20E-02/-4.48E-02/4.44E-03/-2.07E-03/-1.96E-02/1.12E-02/-1.58E-04/-1.06E-02/-5.15E-03/1.07E-02/5.15E-03/-1.32E-03/-6.45E-03/1.69E-02/7.40E-03/-7.13E-03/4.85E-04/-4.24E-04/-2.69E-04/-5.57E-04/-1.02E-03/-1.73E-03/6.64E-04/-3.05E-03/-7.84E-04/-5.20E-03/-2.53E-03/-1.68E-03/-5.90E-03/6.08E-03/1.22E-03/-2.31E-04/1.87E-04/4.62E-03/1.43E-03/-1.60E-03/-5.25E-03/2.83E-03/2.18E-03/-1.40E-032.02E-03 2.73E-03 -3.52E-04 -1.57E-03 -6.73E-04 -1.27E-03 -3.58E-04 -1.41E-03 -5.47E-06 3.72E-03 -1.61E-03 -1.14E-03 -8.09E-04 3.68E-04 6.73E-04 -2.78E-04 1.36E-03 1.16E-03

***H. galaica*-DA0145:** 1.00E+00/1.04E-17/-4.70E-17/2.65E-01/4.00E-02/2.25E-02/-2.24E-01/7.50E-02/5.34E-02/3.69E-03/1.62E-01/6.73E-02/4.57E-02/-2.02E-03/-7.71E-02/-8.54E-03/5.76E-03/-1.55E-02/-4.64E-02/1.10E-02/2.07E-03/-4.42E-03/-4.71E-02/-3.71E-02/3.32E-03/-8.60E-03/-6.66E-03/4.57E-03/5.27E-04/2.22E-03/-5.72E-03/7.19E-03/9.93E-04/-3.58E-03/1.05E-02/2.42E-02/2.03E-03/-5.48E-03/1.25E-03/3.42E-03/-4.59E-04/-8.03E-03/-3.97E-03/1.14E-02/-1.34E-03/1.20E-03/-5.86E-03/-2.86E-03/-1.08E-03/1.58E-03/-4.09E-03/5.29E-03/-4.62E-04/2.48E-03/2.72E-03/1.78E-03/1.83E-03/-1.18E-03/2.11E-03/6.53E-03/-9.78E-04/-2.66E-036.58E-03 2.42E-04 -1.08E-03 -3.41E-03 2.51E-04 3.11E-03 -1.74E-03 -4.79E-05 1.79E-03 2.88E-04 -3.59E-04 1.76E-03 4.15E-04 1.53E-03 5.75E-04 1.00E-03 3.56E-03 9.33E-04

***H. galaica*-DA0146:** 1.00E+00/-6.59E-18/-1.09E-17/2.78E-01/2.18E-02/1.95E-04/-1.83E-01/6.45E-02/6.17E-02/-3.84E-03/1.23E-01/6.55E-02/2.95E-02/-1.09E-02/-1.07E-01/-8.78E-03/3.52E-03/-9.51E-03/-4.56E-02/1.57E-02/-7.23E-03/4.75E-04/-2.84E-02/-2.09E-02/-1.36E-03/-1.91E-03/4.46E-03/2.72E-02/-4.34E-03/-5.03E-04/5.11E-03/1.22E-02/2.79E-03/-6.68E-03/8.81E-03/1.78E-02/1.02E-03/-4.75E-03/-5.42E-03/6.94E-04/-1.13E-03/8.41E-04/-5.15E-03/1.01E-02/-2.03E-03/3.39E-03/3.59E-03/-3.86E-03/-3.59E-04/6.42E-04/6.77E-03/6.53E-03/2.43E-04/-2.68E-03/8.01E-03/1.10E-03/-4.13E-04/-3.31E-03/3.66E-04/1.67E-03/-3.44E-04/-1.89E-03-1.65E-04 -1.66E-03 -6.98E-04 2.31E-03 -9.50E-04 1.77E-03 -2.62E-04 1.68E-03 4.80E-03 -1.75E-03 -7.51E-04 -1.10E-04 2.20E-03 -4.01E-04 -1.32E-03 -2.13E-03 2.95E-03 -5.11E-04

***H. lineolata*-DA0090:** 1.00E+00/-7.88E-18/-1.01E-17/1.97E-01/-4.23E-03/4.42E-03/-3.47E-02/6.15E-02/7.56E-02/-1.43E-02/2.34E-02/2.69E-02/-9.94E-03/-9.20E-03/-1.53E-01/6.89E-03/1.39E-02/-7.22E-03/-1.37E-

02/1.39E-02/-4.52E-03/-5.50E-03/-1.81E-02/-2.42E-03/1.84E-03/-2.96E-03/1.10E-04/1.71E-02/-6.70E-03/-6.52E-03/-2.37E-03/-1.79E-04/7.08E-03/-5.80E-03/2.21E-03/1.14E-02/-2.77E-03/-2.05E-03/-1.57E-04/9.51E-03/3.02E-03/9.55E-04/5.08E-03/6.69E-03/-8.33E-04/-1.44E-03/-4.22E-03/-3.22E-03/9.66E-04/6.64E-04/2.41E-03/3.22E-03/-1.38E-03/-2.40E-03/-3.02E-03/1.01E-03/4.68E-05/-1.67E-03/2.64E-03/3.88E-03/-7.41E-04/-2.03E-032.50E-03 -1.78E-03 2.03E-04 -9.40E-05 3.30E-04 2.21E-03 -1.90E-03 -1.28E-03 2.27E-04 -3.17E-04 3.87E-04 3.54E-05 2.77E-03 2.72E-03 -1.34E-03 -3.57E-04 6.27E-04 -4.33E-04

***H. lineolata* -DA0091:** 1.00E+00/-3.91E-17/1.69E-16/2.27E-01/-2.18E-02/-1.74E-02/1.16E-01/8.58E-02/9.31E-02/-2.28E-02/-1.38E-01/4.58E-02/-1.70E-02/-6.21E-03/-7.06E-02/1.30E-02/3.36E-02/-3.85E-03/-1.39E-03/1.47E-02/-2.45E-02/-9.82E-03/-2.09E-02/-9.00E-04/9.82E-03/-1.00E-02/4.40E-03/8.61E-03/-5.73E-03/-5.32E-03/4.89E-03/1.56E-02/6.25E-03/-4.33E-03/4.45E-03/5.59E-03/-6.25E-03/-1.29E-03/4.43E-03/3.43E-03/4.45E-03/1.14E-03/1.03E-02/8.28E-03/-6.21E-03/-3.57E-03/1.07E-03/-2.14E-04/2.99E-03/1.14E-03/4.24E-03/9.29E-04/-3.96E-03/3.78E-04/4.33E-04/-2.71E-03/7.75E-04/-6.60E-04/2.30E-03/-1.19E-03/-3.90E-03/-9.03E-049.59E-04 -2.66E-03 1.23E-03 3.41E-04 2.33E-03 -2.38E-04 -1.68E-03 -9.59E-04 1.41E-03 -2.55E-03 5.57E-04 1.47E-03 6.29E-04 2.45E-04 -1.30E-03 6.50E-04 9.77E-04 -9.66E-04

***H. lineolata*-DA0092:** 1.00E+00/-1.83E-17/4.61E-17/2.22E-01/-3.24E-02/-2.05E-03/4.78E-02/6.02E-02/8.85E-02/-2.63E-02/-7.86E-02/2.82E-02/-1.82E-02/-7.92E-03/-1.41E-01/1.14E-02/2.39E-02/-6.49E-03/1.27E-02/1.01E-02/-1.63E-02/-6.65E-03/-2.19E-02/9.50E-03/4.64E-04/-6.05E-03/1.06E-02/5.80E-03/-1.80E-03/-4.81E-03/-5.36E-03/9.14E-03/6.61E-03/-1.96E-03/1.32E-02/1.57E-02/-2.19E-03/-3.30E-03/-2.73E-03/1.27E-03/3.06E-03/1.27E-03/3.16E-03/5.99E-03/-3.11E-03/-5.03E-04/-1.64E-03/3.69E-04/9.61E-04/-1.48E-03/3.57E-03/2.36E-03/-2.88E-03/-2.16E-03/2.80E-04/-1.07E-04/1.59E-05/-1.25E-03/2.48E-03/1.24E-03/-2.44E-03/-1.92E-031.52E-03 7.16E-04 8.86E-04 -2.74E-04 3.08E-03 -2.70E-04 -1.76E-03 -1.13E-03 -2.72E-04 -8.85E-04 5.21E-04 2.07E-04 1.58E-03 4.16E-04 -1.50E-03 -2.52E-04 6.68E-05 -8.66E-04

***H. lineolata*-DA0093:** 1.00E+00/-3.48E-18/-4.81E-17/2.29E-01/-3.04E-02/-2.51E-02/4.56E-02/4.33E-02/7.44E-02/-1.34E-02/-3.98E-02/8.16E-02/-1.82E-02/-2.51E-02/-1.62E-01/1.45E-02/1.55E-02/7.62E-03/1.39E-02/3.27E-02/-1.36E-02/-1.81E-02/-9.05E-03/2.74E-03/-6.89E-03/4.26E-03/7.02E-03/1.07E-02/-2.52E-03/-1.40E-02/8.95E-03/7.93E-03/9.06E-03/4.72E-03/1.16E-02/4.67E-03/-5.44E-03/-5.43E-03/9.91E-03/-2.46E-04/2.69E-03/3.97E-03/9.50E-04/2.89E-03/-2.02E-03/-3.89E-03/4.47E-03/1.39E-03/-2.53E-03/1.32E-03/-2.16E-04/3.33E-03/-2.38E-04/-1.68E-03/1.52E-04/-6.12E-03/-2.51E-03/2.37E-03/1.02E-03/-4.04E-04/-6.42E-04/-1.37E-03-2.06E-04 -4.52E-03 -2.34E-03 2.28E-03 3.93E-03 -2.06E-03 1.25E-03 -1.83E-03 6.02E-04 -2.50E-03 -1.58E-03 2.33E-03 -1.64E-03 -7.79E-04 9.78E-04 -5.08E-04 1.02E-03 1.15E-03

***H. lineolata* -DA0094:** 1.00E+00/-3.36E-17/1.30E-16/2.17E-01/-2.08E-02/-2.82E-03/-8.04E-04/5.46E-02/6.80E-02/-1.69E-02/2.23E-02/4.31E-02/-5.82E-03/-1.98E-02/-1.48E-01/1.20E-02/1.23E-02/-4.65E-03/-1.10E-02/2.02E-02/-2.72E-03/-1.61E-02/-2.48E-02/9.72E-04/-2.16E-03/-2.36E-04/1.30E-02/1.13E-02/-7.98E-03/-8.58E-03/-1.22E-03/-6.54E-04/4.80E-03/2.55E-04/6.67E-03/9.58E-03/-3.90E-03/-6.18E-03/5.68E-03/9.00E-03/2.76E-03/-5.66E-04/2.96E-03/7.47E-03/-5.13E-03/-2.22E-03/8.67E-04/-8.81E-04/1.39E-03/2.43E-03/9.43E-04/4.00E-03/-1.60E-03/-6.39E-04/-3.12E-04/-4.24E-03/-2.21E-03/-1.09E-03/2.26E-03/3.06E-03/-1.29E-03/-7.14E-041.22E-03 -2.48E-03 -9.22E-04 8.70E-04 5.50E-03 6.07E-04 -7.97E-04 -1.08E-03 7.11E-04 -2.23E-03 -7.97E-04 2.52E-03 4.53E-04 9.63E-04 -1.24E-04 -2.31E-04 1.78E-03 -7.06E-04

***H. lineolata*-DA0095:** 1.00E+00/3.98E-17/-1.10E-16/2.46E-01/1.76E-02/-3.45E-03/3.89E-02/6.22E-05/7.20E-02/1.08E-02/1.86E-03/4.71E-02/-1.55E-02/-1.95E-02/-1.31E-01/3.05E-02/2.61E-02/5.22E-03/2.35E-03/1.81E-02/-1.39E-02/-1.64E-02/-3.00E-02/1.47E-02/3.49E-03/1.15E-03/-6.50E-03/1.32E-02/-3.07E-03/-4.49E-03/3.64E-04/-4.25E-03/3.28E-04/-1.88E-03/-1.13E-03/1.49E-02/-9.63E-04/-1.85E-03/6.13E-03/-4.40E-03/1.08E-03/1.15E-04/9.66E-03/1.26E-02/-2.21E-03/-4.86E-03/-1.41E-03/-1.30E-03/2.92E-05/1.52E-03/3.86E-03/6.26E-03/3.83E-04/-2.31E-03/2.36E-03/2.97E-03/-1.38E-03/-9.18E-04/-1.48E-03/-9.82E-04/-1.14E-04/-9.72E-043.73E-03 -9.03E-04 -5.74E-04 9.96E-04 -1.10E-03 1.88E-03 -9.25E-04 -1.76E-03 4.90E-04 -4.13E-03 -6.49E-04 1.71E-03 3.28E-03 1.23E-03 -9.34E-05 -8.08E-04 6.80E-04 -2.85E-03

***H. lineolata*-DA0096:** 1.00E+00/4.29E-17/-8.04E-17/2.09E-01/8.64E-03/3.81E-03/1.03E-02/3.19E-02/7.57E-02/4.52E-03/5.39E-02/4.25E-02/-1.36E-02/-2.48E-02/-1.52E-01/1.26E-02/2.00E-02/1.42E-03/-1.92E-02/2.72E-02/-7.55E-03/-1.32E-02/-1.88E-02/5.81E-03/3.43E-03/-3.25E-03/-1.07E-02/1.10E-02/-7.95E-03/-1.26E-03/-2.83E-03/-6.79E-03/3.36E-03/-4.16E-03/-1.81E-03/1.29E-02/-1.96E-03/-3.16E-03/2.07E-03/-4.97E-04/6.28E-04/-1.40E-03/1.23E-02/1.12E-02/-2.01E-03/-3.53E-03/-3.03E-03/9.05E-04/1.20E-03/2.48E-03/7.54E-04/3.35E-03/-2.48E-03/-2.61E-03/4.17E-03/2.24E-03/1.19E-03/6.57E-04/-2.38E-03/3.92E-03/-3.33E-03/-1.93E-034.17E-03 -3.02E-03 5.16E-04 1.25E-04 -1.22E-03 1.56E-03 -1.19E-03 -2.95E-04 1.77E-03 -2.68E-03 -5.87E-04 -3.86E-04 2.91E-03 9.72E-04 -6.24E-04 -9.59E-04 1.77E-03 -8.97E-04

***H. lineolata*-DA0114:** 1.00E+00/-2.64E-17/2.88E-17/2.24E-01/-1.42E-02/-1.39E-02/1.29E-02/4.84E-02/8.06E-02/-1.46E-02/3.99E-02/7.08E-02/-1.60E-02/-1.57E-02/-1.70E-01/2.26E-02/1.68E-02/-1.98E-03/-1.60E-02/2.52E-02/-7.92E-03/-9.79E-03/-8.90E-03/5.49E-03/1.55E-03/-1.35E-03/2.48E-03/1.66E-02/-1.35E-02/-7.46E-03/4.72E-03/7.47E-04/5.61E-03/-6.76E-04/8.52E-03/8.74E-03/2.52E-04/-2.42E-03/9.37E-03/-1.20E-03/9.53E-04/-1.09E-03/9.99E-03/9.62E-03/-8.70E-04/-1.97E-03/-4.45E-03/-1.80E-03/2.72E-04/-9.14E-04/5.23E-03/5.19E-03/-2.20E-03/7.43E-04/1.92E-03/-3.05E-03/-7.61E-04/9.04E-04/-1.79E-04/2.38E-03/-2.61E-03/-1.05E-042.47E-03 -5.35E-03 4.43E-04 1.61E-03 -1.95E-03 -1.37E-04 -5.18E-04 -2.29E-04 3.53E-03 -2.36E-03 -2.92E-04 8.07E-04 1.90E-03 -7.34E-04 3.32E-04 -2.93E-04 -2.44E-04 -1.98E-03

***H. lineolata*-DA0119:** 1.00E+00/6.65E-18/-2.34E-17/1.95E-01/-3.41E-02/-2.03E-02/-3.45E-02/3.84E-02/7.48E-02/-6.26E-03/3.64E-02/6.49E-02/-2.18E-02/-1.48E-02/-1.58E-01/1.92E-02/1.02E-02/4.48E-04/-2.59E-02/2.30E-02/-8.66E-03/-1.03E-02/-2.96E-03/5.72E-03/2.09E-03/3.23E-03/4.63E-03/1.99E-02/-1.11E-02/-7.20E-03/3.93E-03/-4.89E-04/6.55E-03/-5.66E-04/9.36E-03/8.07E-03/-2.45E-03/-2.79E-03/7.00E-03/2.64E-03/3.00E-04/8.08E-04/9.12E-03/9.09E-03/-1.91E-03/-2.92E-03/-2.59E-03/-3.42E-03/-1.64E-03/5.97E-04/-1.66E-04/3.23E-03/-1.95E-03/-9.50E-04/1.27E-04/-3.07E-03/-2.14E-03/1.06E-03/1.31E-03/2.11E-03/-1.89E-03/-1.49E-032.85E-03 -3.23E-03 -1.24E-03 1.36E-03 1.48E-03 -6.17E-04 -1.20E-05 -5.90E-04 3.19E-03 -1.30E-03 -3.50E-04 1.98E-03 2.32E-03 2.14E-04 3.91E-04 -8.61E-04 1.02E-03 -2.55E-03

***H. lineolata*-DA0186:** 1.00E+00/-1.81E-18/-1.75E-17/2.29E-01/-1.75E-02/-2.14E-02/2.39E-02/5.27E-02/8.35E-02/-6.23E-03/1.22E-02/5.45E-02/-1.93E-02/-1.65E-02/-1.56E-01/6.16E-03/2.33E-02/6.85E-04/-7.02E-03/1.87E-02/-1.17E-02/-1.14E-02/-6.68E-03/8.50E-03/1.93E-03/-2.04E-03/-6.21E-03/1.98E-02/-1.02E-02/-7.61E-03/5.65E-03/-2.31E-03/8.07E-03/-1.21E-03/5.34E-03/1.12E-02/-2.46E-03/-5.61E-03/8.16E-03/4.67E-03/1.71E-03/1.05E-03/7.30E-03/4.62E-03/-8.58E-04/-6.50E-03/-1.48E-03/5.33E-04/1.16E-03/3.67E-03/6.15E-03/-2.37E-05/-2.67E-03/-3.26E-03/5.52E-04/3.00E-04/-7.98E-04/7.16E-04/-2.10E-04/2.07E-04/-1.09E-03/-3.04E-031.74E-03 -3.25E-03 -1.42E-03 1.48E-03 6.04E-04 1.98E-03 -4.45E-04 -8.05E-04 1.62E-03 -3.18E-03 -1.09E-03 1.17E-03 2.08E-03 -9.86E-04 -6.08E-04 -2.55E-04 1.10E-04 1.22E-04

***H. lineolata*-DA0187:** 1.00E+00/-1.86E-17/8.53E-17/2.15E-01/2.96E-02/2.42E-03/-7.81E-02/2.16E-02/5.88E-02/1.80E-03/7.79E-02/4.99E-02/-1.95E-03/-4.46E-03/-1.51E-01/1.24E-02/2.76E-03/-2.71E-03/-3.39E-02/3.47E-02/-1.57E-03/-5.19E-03/-1.33E-02/-4.43E-03/-9.53E-04/-3.05E-03/-7.06E-03/2.41E-02/-6.15E-03/-8.45E-04/-6.06E-03/-1.37E-03/-3.32E-04/-3.92E-03/3.43E-03/9.67E-03/4.93E-03/-2.60E-03/7.63E-04/5.78E-04/-4.54E-04/-1.78E-03/8.59E-03/1.01E-02/9.02E-04/-2.31E-03/2.90E-03/-9.84E-04/2.48E-03/-1.78E-03/-2.70E-03/5.80E-03/-7.48E-04/-1.54E-03/4.16E-03/-1.75E-03/-4.39E-04/-1.50E-03/-4.08E-03/5.45E-03/-1.17E-03/-1.13E-033.80E-03 3.48E-04 -6.55E-04 -1.45E-04 2.08E-03 1.56E-04 -1.54E-03 -9.57E-04 2.37E-04 -1.26E-03 -5.96E-04 2.24E-04 -4.81E-04 2.09E-04 -6.69E-04 -1.01E-03 5.21E-03 5.58E-04

***H. lineolata*-BM747067:** 1.00E+00/7.10E-18/-6.06E-17/2.17E-01/-2.09E-02/4.02E-03/-1.47E-02/5.01E-02/8.38E-02/-8.49E-03/4.15E-02/5.37E-02/-1.78E-02/-2.37E-02/-1.43E-01/2.19E-02/1.22E-02/-7.09E-03/-2.45E-02/2.66E-02/-8.05E-03/-3.91E-03/-1.10E-02/1.77E-03/3.63E-03/-2.54E-03/2.35E-03/1.56E-02/-8.95E-03/-7.27E-03/-1.04E-03/-7.87E-03/6.71E-04/-2.04E-03/6.94E-03/1.29E-02/-7.59E-04/-5.97E-04/4.25E-03/5.54E-03/3.08E-03/-8.19E-04/1.22E-02/7.75E-03/-3.93E-03/-4.85E-03/-5.87E-04/-6.43E-04/-7.21E-04/2.38E-03/-1.80E-03/6.33E-03/-2.04E-03/-4.46E-04/2.60E-03/1.95E-04/1.07E-03/-4.83E-04/1.93E-04/-3.77E-04/-3.68E-03/-2.34E-032.23E-03 -4.51E-03 -1.72E-03 9.01E-04 -6.83E-04 1.60E-03 -1.37E-04 -1.64E-05 1.79E-03 -1.55E-03 -6.77E-05 -1.70E-04 3.39E-03 -2.98E-04 -1.00E-03 -1.98E-03 3.53E-04 -1.06E-03

***H. pseudocinerascens*-DA0149:** 1.00E+00/-1.92E-17/8.53E-17/2.75E-01/3.19E-02/1.93E-02/-1.59E-01/4.81E-02/5.25E-02/-1.29E-02/1.42E-01/9.54E-02/2.59E-02/-6.80E-03/-8.29E-02/-1.01E-02/2.07E-03/-1.61E-02/-5.62E-02/2.23E-02/-4.57E-03/-1.06E-02/-3.37E-02/-2.58E-02/3.02E-03/-7.40E-03/-9.09E-03/1.45E-02/-5.56E-03/-7.13E-03/9.98E-03/1.17E-03/-1.14E-03/-4.14E-03/8.30E-03/1.78E-02/-5.65E-04/-1.08E-02/4.77E-03/-2.39E-04/-2.21E-03/-5.34E-04/-1.95E-03/7.61E-03/9.14E-04/-1.74E-03/1.77E-03/3.21E-04/-1.46E-03/2.20E-03/-1.01E-03/6.45E-03/-1.41E-03/7.04E-04/5.32E-03/1.73E-03/-1.98E-03/7.95E-04/9.88E-04/1.60E-03/-1.87E-03/-2.32E-034.11E-03 -1.96E-03 -3.05E-03 -6.26E-05 -1.79E-03 -2.60E-03 -1.70E-03 8.16E-04 2.18E-03 1.38E-03 -1.51E-03 1.21E-04 1.88E-04 -1.87E-04 -7.87E-04 3.17E-04 2.42E-03 1.48E-04

***H. pseudocinerascens*-DA0150:** 1.00E+00/-1.20E-17/4.04E-17/2.68E-01/-2.52E-02/-1.90E-02/-1.19E-01/4.71E-02/7.38E-02/-1.75E-03/1.04E-01/8.62E-02/2.02E-02/-2.39E-02/-9.81E-02/-9.67E-03/1.79E-02/-4.06E-03/-4.34E-02/-9.97E-03/-2.06E-03/-1.79E-02/-3.21E-02/-1.78E-02/1.78E-03/8.98E-04/3.92E-03/1.62E-02/-4.30E-03/-1.24E-02/9.11E-03/1.39E-02/2.83E-03/-3.54E-03/9.81E-03/2.00E-02/3.06E-03/-9.28E-03/-3.57E-03/-1.58E-03/-1.68E-03/1.79E-05/-2.49E-03/5.25E-03/-3.54E-05/-1.76E-03/-2.04E-04/-2.20E-03/-3.41E-03/3.69E-03/2.27E-03/6.64E-03/-1.59E-03/-2.80E-03/4.82E-03/1.17E-03/-2.13E-03/-1.33E-03/8.39E-04/1.16E-03/-1.36E-04/-3.65E-032.48E-03 -3.50E-03 -2.48E-

03 1.00E-03 -1.79E-04 1.90E-03 -3.91E-04 3.24E-04 1.38E-03 1.55E-03 -1.92E-03 1.64E-03 -7.49E-04 1.41E-03 1.39E-04 -1.81E-04 2.47E-03 -1.55E-04

***H. pseudocinerascens*-DA0151:** 1.00E+00/-2.72E-18/2.09E-17/2.32E-01/4.09E-02/2.42E-02/-2.72E-01/8.63E-02/4.33E-02/1.08E-02/1.51E-01/8.80E-02/4.25E-02/-5.37E-03/-8.50E-02/4.45E-03/7.90E-03/-1.38E-02/-4.37E-02/2.84E-03/-4.30E-03/-1.22E-02/-3.69E-02/-3.31E-02/-1.38E-03/-1.09E-02/3.61E-03/4.87E-03/-6.36E-03/5.46E-04/-1.27E-02/8.06E-03/-2.02E-03/-7.80E-04/7.58E-03/3.18E-02/4.30E-03/-5.87E-03/-1.63E-03/1.04E-02/4.06E-03/-5.58E-03/2.99E-03/7.26E-03/-1.94E-03/-4.18E-03/-1.44E-03/-5.84E-03/-4.92E-03/1.76E-03/1.13E-03/4.51E-03/-3.01E-03/2.11E-03/-2.47E-06/-6.08E-07/9.44E-04/1.31E-03/4.86E-03/7.56E-03/1.79E-03/-2.46E-035.61E-03 2.40E-04 2.03E-03 -3.01E-03 1.78E-03 2.76E-03 -1.36E-03 -1.26E-03 2.09E-03 -2.37E-03 -1.37E-03 8.11E-04 8.80E-04 1.65E-03 -1.21E-03 1.57E-03 1.27E-03 -2.21E-03

***H. rugulosa*-DA0140:** 1.00E+00/-1.95E-17/1.29E-17/2.91E-01/2.85E-02/-1.49E-02/-1.75E-01/-3.29E-04/5.59E-02/1.10E-03/1.08E-01/1.14E-01/2.53E-02/-7.05E-03/-7.91E-02/6.77E-03/1.35E-02/-1.89E-03/-3.59E-02/2.14E-02/-3.75E-04/-2.18E-02/-2.74E-03/-3.78E-02/5.78E-03/-4.16E-03/-1.36E-02/-5.69E-03/-5.22E-03/-1.05E-02/-8.82E-03/1.52E-03/-4.75E-03/-1.36E-03/-6.12E-03/2.68E-02/4.20E-03/-5.19E-03/2.32E-03/1.06E-02/-1.88E-03/-2.89E-03/2.28E-03/8.86E-03/1.29E-03/-3.51E-03/9.58E-03/-2.53E-03/-2.97E-03/-6.49E-04/-3.23E-03/-1.39E-03/-1.92E-03/-2.85E-03/1.59E-03/4.20E-03/-3.41E-03/-7.21E-04/-1.27E-03/8.46E-04/1.77E-04/-1.72E-044.22E-03 1.48E-03 9.08E-05 -2.68E-04 2.32E-03 2.11E-04 1.14E-03 9.66E-04 3.61E-03 1.74E-03 -4.63E-04 -2.15E-03 4.79E-04 -8.78E-04 -1.10E-03 3.04E-04 9.53E-04 1.90E-03

***H. rugulosa*-DA0141:** 1.00E+00/8.44E-18/-3.34E-17/2.58E-01/1.28E-02/9.34E-03/-2.24E-01/3.21E-02/6.54E-02/1.53E-02/9.71E-02/9.63E-02/3.41E-02/7.31E-03/-9.82E-02/-3.56E-03/2.16E-02/-3.83E-03/-1.22E-02/2.79E-03/7.71E-03/-1.02E-02/1.70E-02/-4.78E-02/1.39E-02/-1.06E-02/3.24E-03/-1.50E-04/-2.54E-03/-1.02E-02/-2.91E-02/-5.90E-04/5.21E-03/-1.70E-03/-1.95E-02/2.47E-02/6.91E-05/-4.83E-03/-5.69E-03/7.09E-03/2.01E-03/4.11E-03/4.00E-04/7.77E-03/4.65E-04/-5.27E-03/8.80E-03/-1.10E-03/2.37E-03/-3.32E-03/-9.66E-04/5.40E-03/7.81E-04/-7.56E-03/-9.70E-04/1.23E-03/-2.89E-03/-1.12E-03/-6.04E-04/2.50E-03/-5.06E-04/2.38E-043.45E-03 -1.61E-03 -1.44E-04 2.25E-03 -3.46E-03 1.12E-03 2.09E-03 -1.96E-03 -1.64E-03 2.59E-03 -8.66E-05 -1.32E-03 -2.86E-03 2.67E-03 6.64E-04 -2.27E-03 2.41E-03 1.75E-03

***H. rugulosa*-DA0142:** 1.00E+00/2.23E-18/-1.76E-17/2.02E-01/6.97E-02/1.46E-02/-3.20E-01/4.67E-02/3.96E-02/1.92E-02/1.27E-01/8.13E-02/3.61E-02/1.75E-02/-7.20E-02/1.48E-02/1.32E-02/-2.38E-03/-2.02E-02/2.92E-02/3.06E-03/-1.28E-02/-1.30E-02/-3.50E-02/6.45E-03/-1.78E-02/9.65E-03/-1.38E-02/-4.54E-03/-7.80E-03/-2.37E-02/-3.69E-03/-5.41E-03/1.35E-03/-1.08E-02/2.37E-02/-4.90E-04/6.77E-04/-5.75E-03/4.09E-03/4.76E-03/-3.47E-03/1.12E-03/1.11E-02/-8.00E-04/-1.32E-03/1.38E-03/-8.62E-04/8.11E-04/-1.43E-03/1.53E-03/6.84E-03/-2.80E-03/-2.14E-03/1.38E-03/-9.13E-05/-1.87E-03/-1.12E-03/-1.27E-03/5.29E-03/-3.48E-03/8.35E-041.13E-03 -1.68E-03 8.52E-04 -6.45E-04 2.86E-04 2.43E-03 -6.78E-04 4.73E-05 3.50E-03 4.14E-04 -3.56E-04 -1.21E-03 -1.04E-03 3.05E-03 -1.07E-03 2.63E-04 6.44E-04 1.48E-03

***P. romana*- BM670857:** 1.00E+00/5.22E-17/-1.58E-16/3.01E-01/-2.59E-02/-2.05E-02/-3.63E-02/6.14E-02/8.75E-02/-5.89E-04/-4.30E-02/5.90E-02/-1.97E-02/-8.47E-03/-7.04E-02/-2.66E-02/1.43E-02/1.86E-03/6.64E-03/3.62E-02/-9.44E-03/-1.04E-02/-8.53E-03/1.39E-02/2.82E-03/-2.53E-04/1.26E-03/1.43E-02/-5.88E-03/-3.09E-03/6.23E-03/3.40E-03/-1.56E-04/4.15E-03/4.98E-03/1.12E-02/-2.35E-03/-4.14E-03/3.95E-03/-1.49E-03/1.59E-04/2.31E-03/4.11E-03/1.86E-03/-1.99E-03/-2.35E-03/2.92E-03/-8.37E-04/-1.18E-03/1.14E-03/3.41E-03/5.42E-04/1.16E-04/-2.22E-03/3.58E-03/-2.44E-03/-9.42E-04/1.14E-03/-8.24E-04/-1.04E-03/4.73E-04/-9.54E-041.69E-03 -2.46E-03 -6.67E-05 1.08E-03 -8.80E-05 -2.49E-03 1.70E-04 -3.09E-04 2.21E-03 -1.73E-03 -2.33E-04 7.88E-04 -1.74E-03 -6.04E-04 1.05E-03 -1.81E-04 1.77E-04 -6.11E-04
