# Supplementary figures and images for: Inferring speciation modes in a clade of Iberian chafers from rates of morphological evolution in different character systems
Source: BMC Evol Biol. 2009 Sep 15;9:234. doi: 10.1186/1471-2148-9-234 (PMC2753572; doi:10.1186/1471-2148-9-234)

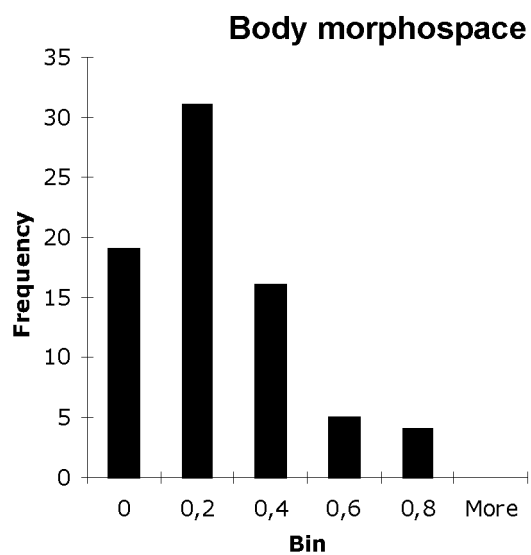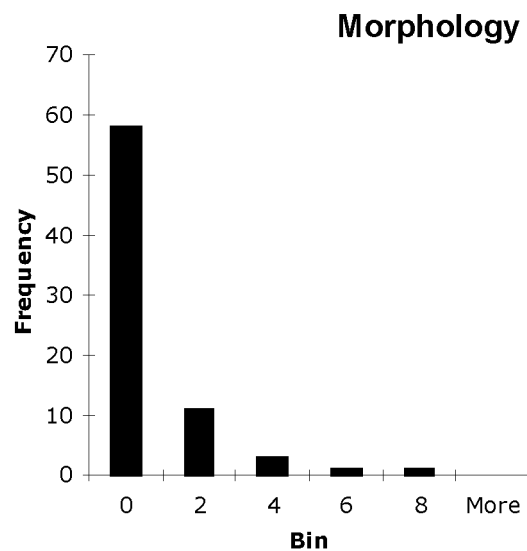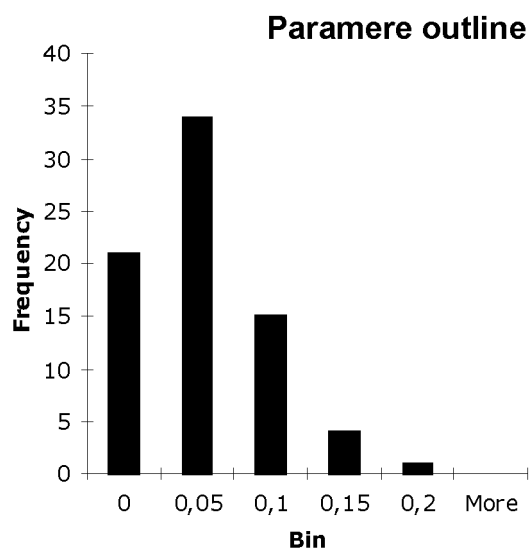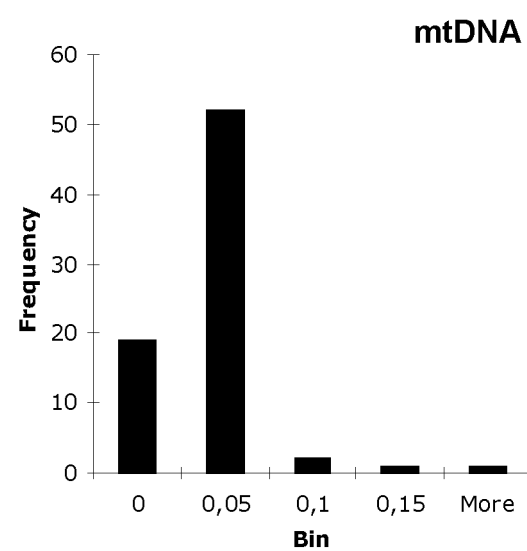

**Additional file 8.** Frequency of branch length distribution of the different traits.

Supplement: Additional file 8 — Frequency of branch length distribution of the different traits: Body-morphospace (above left), morphology (pars, above right), paramere outline shape (below left), mtDNA (below right). Results of the analysis of trait change. [file 1471-2148-9-234-S8.pdf]
