# Supplementary material for: Inferring speciation modes in a clade of Iberian chafers from rates of morphological evolution in different character systems
Source: BMC Evol Biol. 2009 Sep 15;9:234. doi: 10.1186/1471-2148-9-234 (PMC2753572; doi:10.1186/1471-2148-9-234)
Supplement: Additional file 9 — Parsimony (50% majority rule consensus) and consensus phylogram obtained with MrBayes (bootstrap values above 50% and posterior probability are shown below branches). Results of tree searches. [file 1471-2148-9-234-S9.pdf]

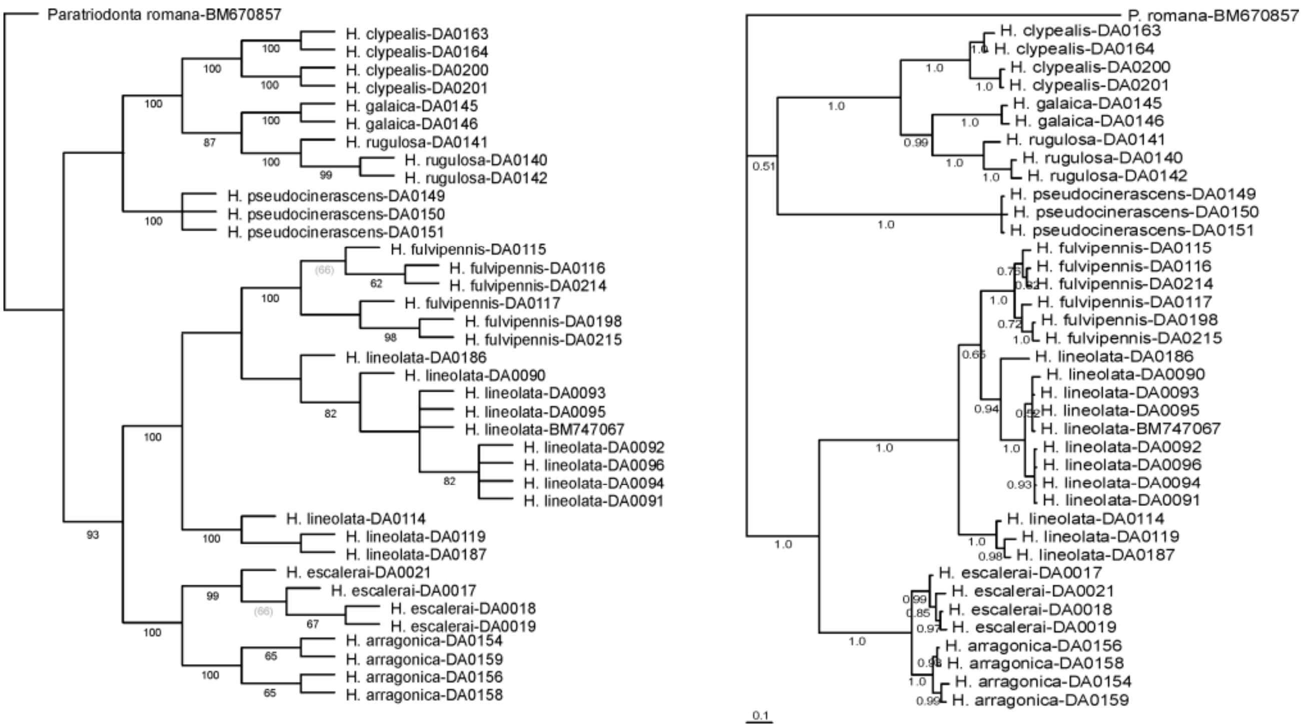

Additional file 9. Parsimony (50% majority rule consensus) and consensus phylogram obtained with MrBayes (bootstrap values above 50% and Posterior probability are shown below branches).
