# Supplementary material for: Inferring speciation modes in a clade of Iberian chafers from rates of morphological evolution in different character systems
Source: BMC Evol Biol. 2009 Sep 15;9:234. doi: 10.1186/1471-2148-9-234 (PMC2753572; doi:10.1186/1471-2148-9-234)
Supplement: Additional file 10 — Plots of Canonical Variate Analysis showing the species-specific divergence in the quantitative morphological traits (body shape and paramere shape; the latter specified for 95%(PC axis 1-22) and 75% (PC axis 1-10) of the variation). Results of Canonical Variate Analysis. [file 1471-2148-9-234-S10.pdf]

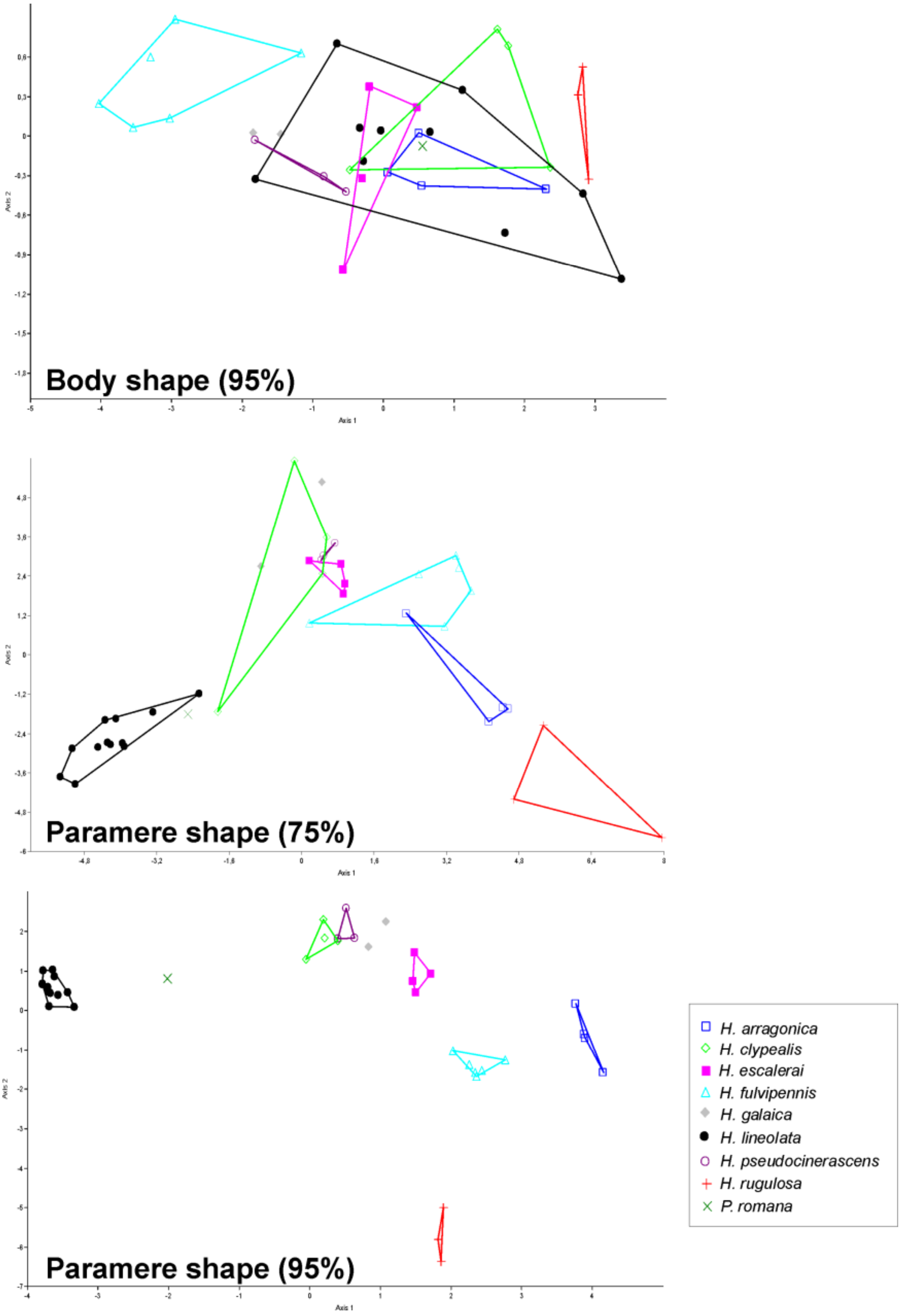

Additional file 10. Plots of Canonical Variate Analysis showing the species-specific divergence in the quantitative morphological traits (body shape and paramere shape; the latter specified for 95%(PC axis 1-22) and 75% (PC axis 1-10) of the variation).
